# Supplementary material for: Implementation science protocol for a participatory, theory-informed implementation research programme in the context of health system strengthening in sub-Saharan Africa (ASSET-ImplementER)
Source: BMJ Open. 2021 Jul 8;11(7):e048742. doi: 10.1136/bmjopen-2021-048742 (PMC8268893; doi:10.1136/bmjopen-2021-048742)
Supplement: Supplementary data [file bmjopen-2021-048742supp001.pdf]

Workshop for ASSET meeting in Addis Ababa Ethiopia, February 2020

Template for identification of contextual and behavioural determinants that can potentially influence the effectiveness of your intervention

A. Context determinant that influence the delivery of care relevant to your work package

Context refers to anything in the environment, that influences the delivery of high-quality, evidence-based care that is person-centred and respectful.

1. List all the contextual barriers/enablers identified in the diagnostic phase of your research that influenced the delivery of high-quality care.
2. For each contextual determinant listed below, theorise the proposed mechanism you think this will influence the implementation of your intervention.
3. We will be visiting each work-package to assist you in any way we can.

Table 1: Contextual determinants that can potentially influence the effectiveness of implementation efforts

Domain	Determinants	Describe how relevant determinant influences the implementation of your intervention
The geographical domain refers to the broader physical environment, landscapes and resources, both natural and transformed by humans, available at a given location. As such, it also comprises the infrastructure at a given location, which	Geography (e.g. altitude, desert, forest, water)<br><br>Climate (e.g. temperature, rainfall)<br><br>Infrastructure (e.g. water and sanitation, energy, transport)<br><br>Access to health care system	i.e. distance to health centre, rainfall, etc

Seward N, *et al. BMJ Open* 2021; 11:e048742. doi: 10.1136/bmjopen-2021-048742

could result in geographical isolation.		
Epidemiological: distribution of disease/conditions, the attributable burden of disease as well as determinants of needs in human populations	Demographics (life expectancy, gender, age, ethnicity, genetic factors)<br><br>Population density, fertility patterns, family size<br><br>Incidence/prevalence and severity of disease, morbidity and mortality	i.e. infectious nature of tuberculosis
Socioeconomic: This domain comprises the economic resources of a community and the access of a population to these resources. It also shows the relationship between an economy and its society.	Social or socio-economic status attributed to education, income, occupation marriage, or gender<br><br>Financial aspects (income, wealth)<br><br>Occupational aspects (employment status, working conditions)<br><br>Living conditions (housing, neighbourhood characteristics)<br><br>Determinants of needs of people directly affected by disease/condition	Example – costs of seeking care and associated childcare costs; lack of education and associated literacy influencing patients ability to seek appropriate care

	Access to health care system	
Sociocultural:<br><br>This domain comprises explicit and implicit behaviour patterns, including their embodiment in symbols and artefacts; the essential core of culture consists of historically derived and selected ideas and values that are shared among members of a group<br><br>This domain not only refers to the conditions in which people are born, grow, live, work and age but also embraces the social roles a human being takes in as a family member, community member or citizen and the relationships inherent to these roles.<br><br>Constructs such as knowledge, beliefs, conceptions, customs, institutions and any	Language and means of communication<br><br>Symbols, heroes, rituals<br><br>Values (e.g. evil vs. good, dirty vs. clean, dangerous vs. safe, abnormal vs. normal)<br><br>Beliefs (e.g. superstition, fate or destiny)<br><br>Religiosity and spirituality<br><br>Knowledge and perceptions (e.g. with respect to significance of health issue, options for resolving health issue)<br><br>Lifestyle (population's patterns in nutrition, smoking, substance abuse)<br><br>Discrimination<br><br>Social capital and resources available through social relationships, specifically	Example – stigma associated with TB, HIV or mental health conditions; acceptance of violence as normal; normalisation of disrespectful care

other capabilities and habits acquired by a group are comprised by this domain	social networks, norms of reciprocity, and trust<br><br>Social cohesion, including relational, material, and political dimensions, information exchange, networks of support, and informal social control<br><br>Historical and contemporary social power relations<br>Sociodemographic profiles<br><br>Psychosocial factors<br><br>Social and societal context<br><br>Structural social inequalities (e.g. Gender inequalities, caste system)<br><br>Community characteristics and level of coordination/involvement with community<br><br>Relevant changes over time (e.g. social changes or social movements, violence)	
Political	Political situation including political stability and absence of violence,	Political uprisings that may results in conflict, that influences the delivery of high-quality care<br>Implementation and support of appropriate guideline-based care

Seward N, *et al. BMJ Open* 2021; 11:e048742. doi: 10.1136/bmjopen-2021-048742

	government effectiveness, voice and accountability, control of corruption, rule of law, regulatory quality, participation, accountability, transparency, efficiency, decency, and fairness<br><br>Health Care System (e.g. governance and leadership, resources, service delivery, integration of patient's needs and perspective)<br><br>Access to health care system<br><br>Relevant changes over time (e.g. political reform*, change of government)	
Legal	Norms, values and beliefs underlying legislation<br><br>Specific legislation (e.g. patient rights, data protection)<br><br>Regulatory provisions concerning healthcare personnel and their rights and duties<br><br>Guidelines	Legislation to protect women from violence; legislation act for mental health

Seward N, *et al. BMJ Open* 2021; 11:e048742. doi: 10.1136/bmjopen-2021-048742

	Decision-making in care delivery<br><br>Sharing of information with indirectly affected stakeholders<br><br>Legislation<br><br>Relevant changes over time (e.g. introduction of new regulation or legislation)	
Ethical	Autonomy<br><br>Moral distress<br><br>Privacy<br><br>Conflicting interests<br><br>Morality and beliefs<br><br>Ethical principles and code of conduct	

B. Characteristics of the organisation implementing the intervention (i.e. primary health centre, hospital, community setting) that influence the delivery of high-quality care

The characteristics of the site where the intervention is going to be implemented is known to influence the effectiveness of implementation efforts.

Below are a list of possible characteristics of the health facility or organisation that can influence the delivery of high-quality care.

In your different work packages, please read the different questions for each of the listed determinants. If you feel the determinants influence the delivery of high-quality care, please theorise and describe the mechanisms by which you feel this occurs.

Determinant	Relevant questions	How does this influence delivery of high-quality care
Structural characteristics: These are objective organisational characteristics – e.g., the size, location, financial turnover, patient volumes, number of services and similar. These characteristics of your hospital may impact positively or negatively on the planned implementation.	How does the infrastructure of the organisation, influence the delivery of high-quality care?<br><br>How will the infrastructure influence the implementation of the intervention?<br><br>Are there any changes in infrastructure that will be required to implement the intervention?	
Networks and communications:<br>Staff and other peer-to-peer networks and communications systems and policies within your organisation. Includes written, oral and other methods of communication.	How available is information to deliver required guideline-based care?<br>How available are people when you need help in solving a problem?<br>How are the working relationships with leaders?<br>Working relationships with influential stakeholders?<br>Working relationships with colleagues?	

Determinant	Relevant questions	How does this influence delivery of high-quality care
Culture: Norms, values, and basic assumptions of a given organization.	How will/does the organisation culture, influence the delivery of high-quality care or the implementation of the intervention?<br>To what extent does the organisation embrace new ideas to improve the organisation?<br>What is the culture like within the health facilities?<br>Are the health facilities innovative?<br>Are the health facilities safety and quality conscious? Or slow to respond?<br>Other cultural elements?	
Implementation climate<br>This is a specific element of organisational culture around how innovations and evidenced interventions tend to get implemented. Check the 6 elements on the left – how do they apply to your organisation?	1. Tension for change<br>2. Compatibility<br>3. Relative priority<br>4. Organisational incentives and rewards<br>5. Goals and feedback<br>6. Learning climate	
Readiness for implementation: As above, this is a cultural element specifically applied to implementing innovations. Check the 3 elements on the right – how do they apply to your organisation?	1. Leadership engagement<br>2. Available resources<br>3. Access to information and technology	
Patients' needs and resources	Is the facility aware of the needs of the patients accessing the health facility?<br><br>Does the facility have the resources to adequately address the needs of the patients?	

Seward N, *et al. BMJ Open* 2021; 11:e048742. doi: 10.1136/bmjopen-2021-048742

Determinant	Relevant questions	How does this influence delivery of high-quality care

C. Determinants of behaviours that influence the delivery of high-quality care

Recognising and targeting determinants of behaviours that influence the delivery of high-quality, person centred care will be an important component of the interventions for the different work packages within ASSET.

The Theoretical Domains Framework (TDF) was developed to identify determinants of specific behaviours. For this exercise we have selected behaviours associate with person-centred care or quality improvement as these are common themes across all work packages.

Once the key determinants for specific behaviours associated with person centred care or quality improvement have been identified using the TDF, there is potential to design interventions that specifically target the identified determinants.

Behaviour change techniques have also been mapped onto the TDF that allows us to identify key determinants for a particular behaviour associated the person-centred care/quality improvement and propose a set of behaviour change techniques.

We ask that each work package splits into two groups – one for person centred care and one for quality improvement. Initially we that you list below the problematic behaviours associated with either person centred care or quality improvement. Once this is complete, we ask that you identify determinants of these behaviours using the template provided in Table 5. Finally, within each work packages, identified problematic behaviours and associated determinants will be cross-checked by individuals on working on the different set of behaviours (i.e. people working on person centred care will cross check quality improvement, and vies versa)

Behaviours associated with person centred care and quality improvement

We have started to list behaviours associated with person-centred care in Table 3 below. Please list any additional behaviours relevant to person centred care that you feel are relevant.

Table 3: Behaviours associated with person-centred care

**Behaviours associated with person centred care**	Indicator details
Person centred communication	Patients are actively involved in discussions and decisions about their care; whether professionals encourage patients and families to express their needs, preferences and concerns; whether professionals monopolise the conversation and the extent to which patients feel engaged and valued.
Supporting self-management	This is the encouragement that professionals provide to help patients understand their central role in managing their condition and making decisions.
Supporting shared decision making	Shared decision-making involves patients and professionals communicating about potential care options, and professionals supporting patients to consider the possible consequences of options and the evidence available before arriving at informed preferences
Respectful, compassionate care	Doctors and nurses show interest in patient as a person and is asked about mental state and/or fear
Empathy, compassion, dignity	Dignity involves ensuring people know they are worthy of respect.<br>Doctors and nurses have empathy with patients' emotions and actual situation

Mapping determinants of behaviours associated with person-centred care or quality improvement

Table five lists potential determinants of problematic behaviours associated with person centred care and the quality improvement. Please work within your groups, to identify determinants of person-centred care or quality improvement for each of the behaviours identified in Table 3 or Table 4.

Seward N, *et al. BMJ Open* 2021; 11:e048742. doi: 10.1136/bmjopen-2021-048742

Table 5: Determinants of behaviours associated with person-centred care

**Determinants of problematic behaviours**	**Examples**	**Is this determinant applicable to the relevant problematic behaviour in your work package**
1.Lack of knowledge<br>(An awareness of the existence of something)	Knowledge (including knowledge of condition/scientific rationale)<br>Procedural knowledge<br>Knowledge of task environment	
2.Lack of skills<br>(An ability or proficiency acquired through practice)	Skills<br>Skills development<br>Competence<br>Ability<br>Interpersonal skills<br>Practice<br>Skill assessment	
3. Social/professional role and identity<br>(A coherent set of behaviours and displayed personal qualities of an individual in a social or work setting)	Professional identity<br>Professional role<br>Social identity<br>Identity<br>Professional boundaries<br>Professional confidence<br>Group identity<br>Leadership<br>Organisational commitment	
4. Beliefs about capabilities<br>(Acceptance of the truth, reality or validity about an ability, talent or facility that a person can put to constructive use)	Self-confidence<br>Perceived competence<br>Self-efficacy<br>Perceived behavioural control<br>Beliefs<br>Self-esteem<br>Empowerment<br>Professional confidence	

**Determinants of problematic behaviours**	**Examples**	**Is this determinant applicable to the relevant problematic behaviour in your work package**
5. Optimism<br>(The confidence that things will happen for the best or that desired goals will be attained)	Optimism<br>Pessimism<br>Unrealistic optimism<br>Identity	
6. Beliefs about Consequences<br>(Acceptance of the truth, reality, or validity about outcomes of a behaviour in a given situation)	Beliefs<br>Outcome expectancies<br>Characteristics of outcome expectancies<br>Anticipated regret<br>Consequents	
7. Reinforcement<br>(Increasing the probability of a response by arranging a dependent relationship, or contingency, between the response and a given stimulus)	Rewards (proximal/distal, valued/not valued, probable/improbable)<br>Incentives<br>Punishment<br>Consequents<br>Reinforcement<br>Contingencies<br>Sanctions	
8. Intentions<br>(A conscious decision to perform a behaviour or a resolve to act in a certain way)	Stability of intentions<br>Stages of change model<br>Transtheoretical model and stages of change	
9. Goals<br>(Mental representations of outcomes or end states that an individual wants to achieve)	Goals (distal/proximal)<br>Goal priority<br>Goal/target setting<br>Goals (autonomous/controlled)<br>Action planning<br>Implementation intention	

**Determinants of problematic behaviours**	**Examples**	**Is this determinant applicable to the relevant problematic behaviour in your work package**
10. Memory, attention and decision processes<br>(The ability to retain information, focus selectively on aspects of the environment and choose between two or more alternatives)	Memory<br>Attention<br>Attention control<br>Decision making<br>Cognitive overload/tiredness	
11. Environmental context and resources<br>(Any circumstance of a person's situation or environment that discourages or encourages the development of skills and abilities, independence, social competence and adaptive behaviour)	Environmental stressors<br>Resources/material resources<br>Organisational culture/climate<br>Salient events/critical incidents<br>Person × environment interaction<br>Barriers and facilitators	
12. Social influences<br>(Those interpersonal processes that can cause individuals to change their thoughts, feelings, or behaviours)	Social pressure<br>Social norms<br>Group conformity<br>Social comparisons<br>Group norms<br>Social support<br>Power<br>Intergroup conflict<br>Alienation<br>Group identity<br>Modelling	
13. Emotion<br>(A complex reaction pattern,	Fear<br>Anxiety	

Seward N, *et al. BMJ Open* 2021; 11:e048742. doi: 10.1136/bmjopen-2021-048742

**Determinants of problematic behaviours**	**Examples**	**Is this determinant applicable to the relevant problematic behaviour in your work package**
involving experiential, behavioural, and physiological elements, by which the individual attempts to deal with a personally significant matter or event)	Affect<br>Stress<br>Depression<br>Positive/negative affect<br>Burn-out	
14. Behavioural regulation (Anything aimed at managing or changing objectively observed or measured actions)	Self-monitoring<br>Breaking habit<br>Action planning	
