# Supplementary material for: Implementation science protocol for a participatory, theory-informed implementation research programme in the context of health system strengthening in sub-Saharan Africa (ASSET-ImplementER)
Source: BMJ Open. 2021 Jul 8;11(7):e048742. doi: 10.1136/bmjopen-2021-048742 (PMC8268893; doi:10.1136/bmjopen-2021-048742)
Supplement: Supplementary data [file bmjopen-2021-048742supp002.pdf]

Documentation of selected HSSIs and associated contextual and intervention determinant

Task: Review the different determinants selected during Thursday's workshop and match to relevant EPOC health system strengthening interventions (attached in email)

Table 1: Identified determinant and associated HSSI/Implementation strategies

Determinant	Health system strengthening intervention/ERIC implementation strategies

Documentation of selected intervention functions and associated determinants of behaviour

Please complete the questions below, each of the identified problematic behaviours identified and the associated TDF determinants.

Table 2:

Question	Description
Name of intervention	
1. What is the behaviour that you want to change?	
2. Why do you want to change it? Why is this a priority?	
3. Who is performing the behaviour?	
4. What do they need to do differently? (In what direction do you want the behaviour to change?)	
5. When do they need to do it?	
6. Where do they need to do it?	
7. How often do they need to do it?	
8. With whom do they need to do it?	
9. Which TDF domains and components are most relevant (give codes)	

Map the determinants of behaviour identified with the TDF, to appropriate intervention functions using the documentation provided

Please see the attached documents to facilitate this process (behaviour change wheel, TDF mapped to BCW)

Table 3: Summary of determinants of behaviours identified with the TDF, associated intervention functions and sources of behaviour

**Determinants of behaviour (TDF)**	**Source of behaviour (COM-B)**	**Intervention function (BCW)**
Knowledge, cognition, memory, behaviour regulation	Capability - psychological	Education, training, enablement
