# Supplementary material for: Implementation science protocol for a participatory, theory-informed implementation research programme in the context of health system strengthening in sub-Saharan Africa (ASSET-ImplementER)
Source: BMJ Open. 2021 Jul 8;11(7):e048742. doi: 10.1136/bmjopen-2021-048742 (PMC8268893; doi:10.1136/bmjopen-2021-048742)
Supplement: Supplementary data [file bmjopen-2021-048742supp003.pdf]

Appendix 3: Topic guide for interviews and focus group discussions with investigators from the work packages

Work package:

Participant name:

Date:

Location

A. Introduction

Interviewer to say his/her name and where they are from.

We are here to get feedback from you on how and why you selected health system strengthening interventions. The information you give us will help us to further understand how HSS interventions are selected.

While we are talking, we are recording what is said with this tape recorder so that we can remember all of the information. There are no right or wrong answers.

B. Interview guides

Interview guide to understand how and why the investigators from the different work packages selected the HSS interventions and implementation outcomes

Objective: to get feedback from the investigators from the different work packages to help understand how and why they selected the health system strengthening interventions and associated implementation outcomes.

[Start tape recording from here]

- **To assess how the HSS interventions were selected**
    - Before you began the pre-implementation phase of your research, did you have in mind what implementation strategies you would use?
    - If this was the case, did these change during the pre-implementation phase? Tell me about the process that caused this change.
    - How (what method was used) to select the HSS interventions? Tell me about the process you went through that enabled you to make the selection.
    - Were stakeholders consulted about potential health system strengthening interventions? If so, what can you describe which stakeholders were involved and how they were involved?
    - Was the Theory of Change workshop used to facilitate the selection of interventions and implementation outcomes? If so, how did you conduct the workshops to do this? If not, why did you think they were not suitable? What did you do instead?
    - Were focus group discussions used to help select the HSS interventions and implementation outcomes? What approach did you take and how did they inform the selection of HSS interventions?

- Were implementation science determinant frameworks used to help select the HSS intervention? Tell me about which ones you used and how you used them to inform the selection. *(Probe for how contextual determinants were identified, role of theory and how used to guide other methods such as ToC or focus groups)*
- Were implementation evaluation frameworks used to help select the HSS interventions? Tell me about which ones you used and how you used them to inform the selection. *(Probe for why and how structure of framework was suitable and how used alongside other methods)*
- Were the implementation outcomes matched with specific HSS interventions? If so, tell me about which outcomes you selected and how the HSS interventions were theorised to impact on those outcomes.
- Was the ERIC tool used to help select the HSS interventions? How was this undertaken?
- Was a literature review done to help inform the selection of HSS interventions and outcomes? In what way did previous evidence inform the selection? What issues did you face in translating previous research to the choice of intervention in your study?
- Did you use any other methods to inform your choice of intervention that we haven't discussed?

Interview guide to finalise the findings from the workshop and to understand if there are any gaps in evidence that will require the review any additional documentation.

Objective: After reviewing the findings from the different work packages on the identified determinants and health system strengthening interventions, undoubtedly there will be a few remaining questions. The purpose of this part of the interview is to raise any remaining questions and answer any questions/concerns the work packages may have.

- **We have the following questions about the determinants and HSS interventions that you have selected:**

- **Do you have any remaining questions/concerns about your selection of HSS interventions and implementation outcomes?**

[Stop tape recording when completed]
