# Supplementary material for: Implementation science protocol for a participatory, theory-informed implementation research programme in the context of health system strengthening in sub-Saharan Africa (ASSET-ImplementER)
Source: BMJ Open. 2021 Jul 8;11(7):e048742. doi: 10.1136/bmjopen-2021-048742 (PMC8268893; doi:10.1136/bmjopen-2021-048742)
Supplement: Author's manuscript [file bmjopen-2021-048742.draft_revisions.pdf]

BMJ Open

BMJ Open is committed to open peer review. As part of this commitment we make the peer review history of every article we publish publicly available.

When an article is published we post the peer reviewers' comments and the authors' responses online. We also post the versions of the paper that were used during peer review. These are the versions that the peer review comments apply to.

The versions of the paper that follow are the versions that were submitted during the peer review process. They are not the versions of record or the final published versions. They should not be cited or distributed as the published version of this manuscript.

BMJ Open is an open access journal and the full, final, typeset and author-corrected version of record of the manuscript is available on our site with no access controls, subscription charges or pay-per-view fees (http://bmjopen.bmj.com).

If you have any questions on BMJ Open's open peer review process please email info.bmjopen@bmj.com

BMJ Open

Implementation Science Protocol for a participatory, theory-informed implementation research programme in the context of health system strengthening in sub-Saharan Africa (ASSET-ImplementER)

Journal:	*BMJ Open*
Manuscript ID	bmjopen-2021-048742
Article Type:	Protocol
Date Submitted by the Author:	06-Jan-2021
Complete List of Authors:	Seward, Nadine; King's College London, Centre for Implementation Science, Department of Health Services and Population Research<br>Murdoch, Jamie; University of East Anglia, School of Health Sciences<br>Hanlon, Charlotte; King's College London, Institute of Psychiatry, Psychology and Neuroscience, Health Service and Population Research Department, Centre for Global Mental Health; Addis Ababa University College of Health Sciences, School of Medicine, Department of Psychiatry, WHO Collaborating Centre for Mental Health Research and Capacity-Building<br>Araya, Ricardo; Centre for Global Mental Health and Primary Care Research,<br>Gao, Wei; King's College London, Palliative Care, Policy & Rehabilitation<br>Harding, Richard; King's College London, Dept of Palliative Care and Policy<br>Lund, Crick; King's College London, Centre for Global Mental Health, Department of Health Service and Population Research Department; University of Cape Town, Alan J Flisher Centre for Public Mental Health, Department of Psychiatry and Mental Health<br>Hinrichs-Krapels, Saba; King's College London, The Policy Institute; King's College London, King's Global Health Institute<br>Mayston, Rosie; King's College London, King’s Global Health Institute; King's College London, Global Health and Social Medicine<br>Kartha, Muralikrishnan ; King's College London, King's Health Economics<br>Prince, Martin; King's College London, King's Global Health Institute<br>Sandall, Jane; King's College London, Women and Children’s Health, School of Life Course Sciences<br>Thornicroft, Graham; King's College London, Centre for Implementation Science, Department of Health Services and Population Research; King's College London, Centre for Implementation Science, Health Services and Population Research<br>Verhey, Ruth ; Friendship Bench Zimbabwe,<br>Sevdalis, Nick; King's College London, Centre for Implementation Science, Department of Health Services and Population Research
Keywords:	PUBLIC HEALTH, STATISTICS & RESEARCH METHODS, TROPICAL MEDICINE

SCHOLARONE™
Manuscripts

I, the Submitting Author has the right to grant and does grant on behalf of all authors of the Work (as defined in the below author licence), an exclusive licence and/or a non-exclusive licence for contributions from authors who are: i) UK Crown employees; ii) where BMJ has agreed a CC-BY licence shall apply, and/or iii) in accordance with the terms applicable for US Federal Government officers or employees acting as part of their official duties; on a worldwide, perpetual, irrevocable, royalty-free basis to BMJ Publishing Group Ltd ("BMJ") its licensees and where the relevant Journal is co-owned by BMJ to the co-owners of the Journal, to publish the Work in this journal and any other BMJ products and to exploit all rights, as set out in our licence.

The Submitting Author accepts and understands that any supply made under these terms is made by BMJ to the Submitting Author unless you are acting as an employee on behalf of your employer or a postgraduate student of an affiliated institution which is paying any applicable article publishing charge ("APC") for Open Access articles. Where the Submitting Author wishes to make the Work available on an Open Access basis (and intends to pay the relevant APC), the terms of reuse of such Open Access shall be governed by a Creative Commons licence – details of these licences and which Creative Commons licence will apply to this Work are set out in our licence referred to above.

Other than as permitted in any relevant BMJ Author's Self Archiving Policies, I confirm this Work has not been accepted for publication elsewhere, is not being considered for publication elsewhere and does not duplicate material already published. I confirm all authors consent to publication of this Work and authorise the granting of this licence.

For peer review only

Implementation Science Protocol for a participatory, theory-informed implementation research programme in the context of health system strengthening in sub-Saharan Africa (ASSET-ImplementER)

Nadine Seward[1], Jamie Murdoch[2], Charlotte Hanlon [3,4,] Ricardo Araya[4], Wei Gao[5], Richard Harding[5], Crick Lund[4,6], Saba Hinrichs-Krapels[8,9], Rosie Mayston[9], Muralikrishnan R. Kartha[10], Martin Prince[9], Jane Sandall[11], Graham Thornicroft[1,4], Ruth Verhey[12], Nick Sevdalis[1]

Corresponding author: Dr Nadine Seward, 16 De Crespigny Park, Camberwell, London SE5 8AB, nadine.seward@kcl.ac.uk

Affiliations:

1. Centre for Implementation Science, Health Service and Population Research Department, Institute of Psychiatry, Psychology & Neuroscience, King's College, UK
2. School of Health Sciences, University of East Anglia
3. Department of Psychiatry, WHO Collaborating Centre for Mental Health Research and Capacity-Building, School of Medicine, College of Health Sciences, Addis Ababa University, Ethiopia
4. Centre for Global Mental Health, Health Service and Population Research Department, Institute of Psychiatry, Psychology & Neuroscience, King's College London, UK
5. Florence Nightingale Faculty of Nursing Midwifery and Palliative Care, Cicely Saunders Institute
6. Alan J Flisher Centre for Public Mental Health, Department of Psychiatry and Mental Health, University of Cape Town
7. King's Global Health Institute, King's College London
8. The Policy Institute, King's College London
9. King's Global Health Institute, King's College London, UK
10. King's Health Economics, Kings College London
11. Dept of Women and Children's Health, School of Life Course Sciences, FoLSM, Kings College London
12. Research Support Centre, College of Health Sciences, University of Zimbabwe

For peer review only

Abbreviations
ASSET: Global Research Unit on Health System Strengthening in sub-Saharan Africa
AIM: Acceptability of Intervention Measure
ASSET-ImplementER: Implementation science theme within ASSET
BCW: Behavioural Change Wheel
CFIR framework: Consolidated Framework for Implementation Research
CICI framework: Context and Implementation of Complex Interventions
EPOC taxonomy: Effective Practice and Organisation of Care taxonomy for health system strengthening interventions
ERIC taxonomy: Expert Recommendations for Implementing Change taxonomy of implementation strategies
FIM: Feasibility of Intervention Measure
HSS: Health systems strengthening
IAM: Intervention appropriateness Measure
LMICs: Low- and Middle-Income Countries
NIHR: National Health Institute of Research
STaRI: Standards for Reporting Implementation Studies
TDF: Theoretical Domains Framework
ToC: Theory of Change
WP: Work package

Word count: 4839

Key words: implementation research, implementation science, health systems strengthening, global health, Sub-Saharan Africa

For peer review only

ABSTRACT

Objectives

ASSET (Health System Strengthening in Sub-Saharan Africa) is a health system strengthening (HSS) programme that aims to develop and solutions that support high-quality care. Here we present the protocol for the implementation science (IS) theme within ASSET that aims to (1) understand what HSS interventions work, for whom and how; and (2) how implementation science methodologies can be adapted to improve the HSS interventions within resource-poor contexts.

Settings

Eight work packages in rural and urban areas in publicly funded health facilities in Ethiopia, South Africa, Sierra Leone, and Zimbabwe

Participants

Research staff including principal investigators, co-investigators, field staff, PhD students and research assistants.

Interventions

Pre-implementation phase: The IS theme, jointly with ASSET work-packages, applies IS determinant frameworks to identify factors that influence the effectiveness of delivering evidence-informed care. Determinants are used to select a set of HSS interventions for further evaluation, where work packages also theorise selective mechanisms.

Piloting phase and rolling implementation phase: Work-packages pilot the HSS interventions. An iterative process then begins involving evaluation, refection and adaptation. Throughout this phase, IS determinant frameworks are applied to monitor and identify barriers/enablers to implementation. Selective mechanisms of action are also investigated. In a final workshop, ASSET teams come together, to reflect and explore the utility of the selected IS methods and provide suggestions for future use.

For peer review only - http://bmjopen.bmj.com/site/about/guidelines.xhtml

Structured templates are used to organise and analyse common and heterogeneous patterns across work-packages. Qualitative data are analysed using thematic analysis and quantitative data is analysed using means and proportions.

Conclusions

We use a novel combination of IS methods at a programmatic level to facilitate comparisons of determinants and mechanisms that influence the effectiveness of HSS interventions in achieving implementation outcomes across different contexts. The study also contributes conceptual development and clarification at the underdeveloped interface of implementation science, HSS and global health.

Strengths and limitations of this study

- The purpose of this protocol paper is to describe the methodology for the implementation science theme within ASSET (ASSET-ImplementER).
- The overall aim of the ASSET-ImplementER theme is to advance our understanding of how to design and evaluate HSS interventions using a systems level approach informed by implementation science, across different health systems and contexts.
- This is one of the first large-scale implementation research programmes for health system strengthening in Sub-Saharan Africa that spanning three care platforms (primary care for the integrated treatment of chronic conditions in adults, maternal and newborn care, surgical care), involving eight work packages in four sub-Saharan African countries that aims to apply robust, implementation science and other theory-informed approaches to understand what works for whom and how. Equally as important, we aim to improve implementation science methodologies to design and evaluate HSS interventions within LMIC settings.
- Although our programme is theory-informed through the use of ToC workshops, implementation science determinant frameworks, and exploring how context influences the mechanisms of action introduced by the HSS interventions, we have not used implementation science theories or other middle-range theories to guide the design and evaluate of ASSET as a programme. This is a pragmatic approach given many of the work-packages teams were unfamiliar with implementation science methods at the time of ASSET set-up.
- Although there are limitations to our approach, we expect to begin advance our understanding of what HSS work for whom, and how. It is also hoped that we will start to address the issues in understanding the complexity surrounding how to effectively strengthen health systems in resource-poor contexts within LMICs.

For peer review only - http://bmjopen.bmj.com/site/about/guidelines.xhtml
For peer review only

BACKGROUND

Achieving Universal Health Coverage that includes the availability and delivery of high-quality evidence-informed care has been identified as an urgent priority for health system strengthening in low- and middle-income countries (LMICs).(1, 2) The scale of unmet need is reflected in estimates that that suggest 5 million people in LMICs who engaged with the health system died due to poor-quality health care in 2016, and that 3.6 million deaths occurred due to people not accessing care at all.(3) Improving population health will require not only increased access to health services, but also the provision of high-quality care.(4)

There is lack of consensus as to what constitutes health systems strengthening (HSS).(5) Initially, the term HSS was a reaction to the limitations of vertical programmes targeting specific diseases, that do not strengthen the broader health system.(5) Over time, the definition of HSS changed to one that has system-level effects and not just organisational level effects. It is now recognised that health systems are dynamic and complex socio-technical structures, composed of multiple interacting components that are constantly adapting to changes in the local context and therefore behave unpredictably.(6) HSS to improve quality of care is about "*permanently making the system function better, and not just about filling gaps or supporting the system to produce better short-term outcomes*".(7) HSS involves comprehensive changes to policies and regulations, organisational structures, and relationships across the health system building blocks (e. g. service delivery, health workforce, health information systems, access to essential medicines, financing, and leadership/governance) that motivate changes in behaviour, and/or allow more effective use of resources across multiple care platforms.(6, 8)

Interventions to strengthen health systems, by their very nature, improve health outcomes by providing components that influence several mechanisms, both simultaneously and in isolation, at various time points and at various levels of the health system.(6) As such, HSS requires an approach

For peer review only - http://bmjopen.bmj.com/site/about/guidelines.xhtml

to design and evaluate complex public health interventions, in real-world contexts, that accounts for the multiple interconnecting components and actors.(9) A systems-level approach to the design and evaluation of HSS interventions views a complex intervention as a system in itself, interacting with other building blocks of the underlying health system in which the intervention embeds itself, setting off reactions that may well be unexpected or unpredictable.(6) Applying this approach to the design and evaluation of HSS interventions requires an evaluation of not only their main effects, but also inputs, outputs, initial, intermediate and eventual outcomes, feedback processes and contexts within the underlying health system.(6)

Applying implementation science concepts and methods to health system strengthening

Implementation research is a rapidly expanding discipline that seeks to understand what, why, and how interventions work in real-world settings.(10) Implementation research can consider any aspect of implementation, including understanding contextual and behavioural barriers that influence implementation efforts, the process of implementation, and the evaluation of implementation efforts for outcomes such as reach, fidelity and sustainability.(10, 11) By addressing implementation challenges through the participation of actors from multiple disciplines, implementation research can help to apply a systems-level approach and extract lessons that contribute to building stronger and more resilient and responsive health systems.(12)

Whereas implementation *research* seeks to understand what, why, and how interventions work in real-world settings,(10) implementation science is the study of methods used to carry out this research.(13) Specifically, implementation science offers theories, models, frameworks and other methodologies to optimise and evaluate the implementation of evidence-informed care.(13) The methods offered through implementation science can inform decision-makers on how best to design, implement and adapt HSS interventions in the dynamic environments in which they work, to produce new knowledge for more effective strategies and implementation.(14) For example, implementation

For peer review only - http://bmjopen.bmj.com/site/about/guidelines.xhtml

science determinant frameworks describe contextual and behavioural barriers that are known to influence implementation outcomes, and include key factors to consider in the evaluation of the process of implementation.(15) Box 1 describes key implementation science definitions as used within the ASSET-ImplementER theme.

Box 1: Implementation science terminology used within the ASSET-ImplemenER programme

Implementation strategies: methods or techniques used to enhance the adoption, implementation and sustainability of a clinical program or practice. Other terminology includes HSS interventions, quality improvement strategies.

Implementation outcomes: Implementation outcomes assess how well implementation has occurred or provide insights about how this contributes to an individual's health status or other important health outcomes. Proctor et al articulate the following core set of seven implementation outcomes: acceptability, feasibility, uptake, penetration, cost, fidelity, and sustainability.(16)

Implementation effectiveness: the impact of implementation efforts on implementation outcomes.

Evaluation framework: Specifies implementation outcomes that can be evaluated to determine implementation effectiveness.(17)

Context: Any feature of the circumstances in which an intervention is conceived, developed, implemented and evaluated.(18) Other commonly termed setting or environment.

Determinant frameworks: identify contextual barriers and/or enablers that are known to impact on the effectiveness of implementation efforts.(15)

Theoretical frameworks: identify determinants of behaviours that are known to influence implementation outcomes.(19)

Implementation theories/middle range theories: describe the mechanisms behind how a proposed intervention works. These theories can also be used to identify barriers and/or enablers to change and what needs to change.(15)

Programme theory: describes how a specific intervention is expected to lead to its effects and under what conditions.(20)

Need for improved methods to design and evaluate health system strengthening interventions in LMICs

Despite the need to account for the complexity of HSS interventions, current methods used to design and evaluate such interventions in LMICs typically lack robust methodologies that enable an understanding of what interventions worked, for whom, and how.(21) As an example, few studies to-date have been supported by a conceptual framework (including a programme theory) explaining how an intervention is theorised to work, or a detailed process evaluation that helps to explain whether the intervention worked as intended, and if so, for whom, and under what circumstances.(21-24) There are also limitations with how studies report how the local context influences the effectiveness

For peer review only - http://bmjopen.bmj.com/site/about/guidelines.xhtml

of HSS interventions on important implementation outcomes such as coverage, acceptability and fidelity. Importantly, much of the evidence base for HSS interventions still comes from high-income countries, with uncertain generalisability of evidence to low-resourced settings.(25)

Compounding the methodological shortcoming with HSS in LMICs, are the inconsistences in the terminology used to describe the science of promoting and supporting the use of evidence in health and healthcare policy(26, 27) and also its reporting. As an example, implementation science has been described variously as quality improvement, knowledge translation, knowledge transfer, and even complex intervention evaluation.(27) Although there are differences between fields such implementation science and improvement science, they share the objective of improving the quality of healthcare delivery and therefore clinical outcomes.(28, 29)

Perhaps even more important is the lack of consistency in reporting and describing the implementation strategies to improve the quality of service delivery and therefore clinical outcomes.(30, 31) This results in the inability to replicate or generalise research studies to different contexts, or to allow for research synthesis such as meta-analysis or systematic reviews.(32, 33) To help address this issue, the Expert Recommendations for Implementing Change (ERIC) study created a taxonomy of implementation strategies(32) – to allow researchers to apply a common language when describing how evidenced interventions are being implemented. Taxonomies for quality improvement strategies have also been created that are conceptually similar (in some cases identical) to the ERIC taxonomy.(32, 34) As an example, both taxonomies include audit and feedback strategies, provider education, patient education, patient reminders and organisational change.(32, 34) There is also a taxonomy for HSS interventions, the Effective Practice and Organisation of Care (EPOC) taxonomy, that is also similar to the taxonomies for implementation and quality improvement strategies.(32, 35)

For peer review only

There is, therefore, an urgent need to improve not only the quality of implementation research for HSS in LMICs, but also consistency in reporting the implementation strategies used to deliver evidence-informed care and improve service delivery and patient outcomes. For purposes of the ASSET-ImplementER theme, we will refer to these interventions as HSS interventions. Embedding high-quality implementation science methodology in HSS research that includes consistent reporting of the HSS interventions used to deliver evidence-informed care, will help unpack the 'black box' of how such interventions work (or fail to reach their expected potential) for certain populations in a given setting.(22) The ASSET-ImplementER study aims to start addressing some of these issues.

The National Health Institute of Research (NIHR) Global Research Unit on Health System Strengthening in Sub-Saharan Africa (ASSET) and the ASSET-Implementation Research theme (ASSET-ImplementER)

ASSET is a four-year HSS research programme (2017-21) that addresses the imperative of using implementation research with robust implementation science methods that use consistent reporting of the HSS interventions to deliver high-quality care. ASSET spans three healthcare platforms (primary health care for the integrated treatment of chronic conditions in adults, maternal and newborn, surgical care) involving eight work packages within four different countries within sub-Saharan Africa. The overall aim of ASSET is to develop, implement and evaluate high quality HSS interventions that are effective and sustainable. ASSET also aims to ensure care is equitable, people-centred and respectful.(36) ASSET is one of the first implementation research programmes for HSS that applies an 'effectiveness-implementation hybrid' approach, blending components of study designs that evaluate the effectiveness of interventions on patient outcomes, with study designs that focus on evaluating the effectiveness of HSS interventions on implementation outcomes.(37) This methodology allows ASSET to understand both clinical and implementation effectiveness as well as contextual factors influencing implementation outcomes, including the potential sustainability of the different HSS interventions.

For peer review only - http://bmjopen.bmj.com/site/about/guidelines.xhtml

The ASSET programme includes two phases of implementation research including the pre-implementation phase, and piloting and rolling implementation phase. The *pre-implementation phase* aims to understand requirements for HSS through the evaluation of the structure and function of the health system (such as arrangements for financing, governing, and delivering care, and implementation strategies) (35) that may limit the potential to deliver evidence-informed and person-centred care. At the end of this phase, each work package use research findings to select a set of HSS interventions. The *piloting and rolling implementation phase initially* pilot the interventions to identify factors that influence the implementation of the proposed interventions. After adjusting the initial programme theory and set of HSS interventions, an adaptive and iterative process is used to test the effectiveness of the set of HSS interventions on both clinical and implementation outcomes. The influence of context on the effectiveness of the HSS interventions delivering evidence-informed care is also assessed.(36)

Aim and objectives

The overall aim of the ASSET-ImplementER theme is to advance our understanding of how to design and evaluate HSS interventions using a systems level approach informed by implementation science, across different health systems and contexts. In doing so, we expect to achieve two equally important objectives for HSS in resource-poor contexts in LMICs: (1) advance our understanding of what HSS interventions work, for whom and how; and (2) improve implementation science methodologies to design and evaluate HSS interventions within LMIC settings.

The following specific objectives will be addressed within the ASSET-ImplementER theme:

Pre-implementation and piloting phases

1. Contrast and compare the contextual determinants identified by each work package that influence the effectiveness of evidence-informed care;

For peer review only - http://bmjopen.bmj.com/site/about/guidelines.xhtml

2. Contrast and compare HSS interventions selected by each work package that have the potential to improve the effectiveness of evidence-informed care;
3. Determine how the identified contextual determinants influence ASSET investigators in selecting specific HSS interventions and associated implementation outcomes and compare findings between ASSET work-packages.

Implementation and evaluation phase of ASSET HSS interventions:

4. Contrast and compare findings between the different work-packages on how context influences the delivery of the selected HSS interventions on standardised implementation outcomes measures for acceptability, appropriateness and feasibility;
5. Evaluate the usefulness and utility of the selected implementation science methods/frameworks in achieving a systems level approach in the evaluation of HSS interventions in resource poor contexts and provide suggestions for future applications of implementation science to HSS.

METHODS

Setting

ASSET is working on three care platforms: (1) surgical care; (2) maternal and newborn care, and; (3) integrated primary care, across four sub-Saharan African countries: Ethiopia, Sierra Leone, South Africa, and Zimbabwe. Table 1 describes the ASSET eight work-packages.

For peer review only - http://bmjopen.bmj.com/site/about/guidelines.xhtml

Table 1: Description of the ASSET work-packages for the different healthcare platforms; implementation research cuts across all of them (ASSET-ImplementER theme)

Healthcare platform	Country	Specific work-package (WP)	ASSET-ImplementER
**Primary health care for the integrated treatment of chronic conditions**	Ethiopia	WP1. Primary care for integrated person-centred continuing care of persons with chronic non-communicable diseases including diabetes and hypertension, comorbid with common mental disorders.	
	South Africa	WP4. Promoting person-centred TB care.<br>WP5. Integrated primary palliative care for persons with chronic lung disease.	
	Zimbabwe	WP8. Primary care for integrated treatment of persons with chronic non-communicable diseases including diabetes and hypertension, comorbid with common mental disorders.	
**Maternal and newborn care**	Ethiopia	WP2. Integrated, person-centred and high-quality maternal and newborn care across the antenatal, intrapartum, delivery and neonatal continuum. Psychosocial care for intimate partner violence is nested within this work package.	
	South Africa	WP6. Integrated psychosocial care/support for perinatal women experiencing depression or anxiety or exposed to domestic violence.	
**Surgical care**	Ethiopia	WP3. Increasing access and quality of surgical and dental care.	
	Sierra Leone	WP7. Increasing access to quality, equitable and affordable surgical care.	

Design

The work-packages use mixed-methods throughout ASSET to select HSS interventions in the pre-implementation phase and evaluate the intervention through 'effectiveness-implementation hybrid' designs in the piloting and rolling implementation phase. Hybrid designs are essential with implementation research as they blend the components of study designs used to evaluate clinical effectiveness, with those of implementation study designs that focus on the evaluation of the influence of context on the effectiveness of HSS interventions.(37)

Throughout ASSET, the ASSET-ImplementER stream uses mixed methods including workshops, semi-structured interviews and documentary analyses, to standardise, record and synthesise findings from the implementation component of the different work-packages. Findings include information from

For peer review only - http://bmjopen.bmj.com/site/about/guidelines.xhtml

the different frameworks such as context, intervention, selected HSS interventions, and implementation outcomes. Figure 1 describes the flow of methods for the ASSET-ImplementER theme.

To standardise methods and facilitate cross-site comparisons across ASSET, work-packages select contextual and behavioural determinants and implementation outcomes from a defined set implementation science frameworks. These frameworks are relevant to the programme as a whole, yet at the same time account for the specific characteristics of the different work-packages. Table 2 describes the frameworks that the various work-packages use and how this is relevant to the overall ASSET programme.

Table 2: Selected implementation science frameworks and theories used within ASSET-ImplementER

Framework	Framework type and description	Relevance to ASSET
**Consolidated framework for Implementation Research (CFIR)**	The CFIR is a determinant framework that Includes five domains (inner setting, outer setting, intervention characteristics, characteristics of individuals involved, and the processes of implementation).(38) Within the five domains, are 37 constructs that can behave as a barrier and/or enabler to the implementation of an intervention	The appeal of the CFIR framework is the broad range of constructs that apply not only to individual characteristics, but also structural characteristics of the health care system as well as characteristics of the intervention itself. (38)
**Context and Implementation of Complex Interventions (CICI)**	The CICI is both a determinant and evaluation framework that contains three dimensions (context, implementation and setting) that interact with the intervention. The contextual dimension consists of seven contextual domains (i.e. geographical, epidemiological, socio-cultural, socio-economic, ethical, legal, political) with interact with one another at the micro, meso and macro levels.(39)	The appeal of this framework is the emphasis on the external contextual elements such as sociocultural, socioeconomic and political determinants. These determinants are particularly relevant in low-resource contexts.<br><br>This framework also explicitly addresses complexity required with the design and evaluation of HSS interventions, through the evaluation of the interaction of context (including leadership elements), implementation and setting dimensions with the intervention dimension, at the micro, meso, and macro levels.
**Theoretical Domains Framework (TDF)**	The implementation of evidence-informed interventions is dependent on changing multiple behaviours of different people.(40) where the use of theory to inform behaviour	Many of the barriers to HSS interventions for ASSET are associated with determinants of current and desired behaviours e.g. in health

For peer review only - http://bmjopen.bmj.com/site/about/guidelines.xhtml

	change interventions has been shown to improve the implementation effectiveness.(41) We will adopt the Theoretical Domains Framework (TDF) that represents a synthesis of 128 determinants of behavioural change.(19)	worker or health service users. As an example, social norms that stigmatise common mental health conditions can act as a barrier to respectful, person-centred care. These determinants are particularly relevant to providing person-centred respectful care as well as high quality care.
**Behaviour Change Wheel**	The implementation of evidence-informed practice is dependent on effective behaviour change interventions. The Behavioural Change Wheel is framework that includes nine intervention functions that address determinants of problematic behaviours that require changing.	To ensure longer term sustainability, implementing evidence-informed practice for HSS will involve a degree of behavioural change. Once determinants of the problematic or desirable behaviours have been identified using the TDF, the BCW can be applied to help select appropriate interventions that have demonstrated effectiveness.
**Proctor et al Implementation outcome framework**	An evaluation framework that includes the following eight implementation outcomes: acceptability, adaptability, appropriateness, feasibility, fidelity, cost, penetration, and sustainability.(42)	All of these outcomes are relevant to ASSET at different time points in the implementation of the HSS interventions. As an example, the acceptability, appropriateness and feasibility of the HSS interventions are important to measure in the pre-implementation/piloting phases as they can predict the longer-term sustainability of the intervention. (ref)

The Consolidated Framework of Implementation Research (CFIR) is a determinant framework that is used as it provides an overview of a broad range of determinants that influence implementation effectiveness such as the inner setting (i.e. characteristics of the health facility), characteristics of the intervention (e.g. acceptability, complexity and adaptability) and implementation processes (e.g. regular feedback about progress and quality of implementation).(38) The Context and Implementation of Complex Intervention (CICI) framework is another framework that we use as it offers a detailed approach to identifying determinants from the external context (e.g. sociocultural, socioeconomic, political, epidemiological, ethical, and legal) that are known to influence implementation effectiveness that are particularly relevant to LMICs.(39) It is expected that both of these frameworks will provide a detailed spectrum of determinants that are relevant to the ASSET programme. However, the frameworks are not exhaustive: any determinant identified that is not a part of either frameworks will be documented as such.

For peer review only - http://bmjopen.bmj.com/site/about/guidelines.xhtml

Further to contextual determinants, it is also important to understand in some detail characteristics of the users who deliver the healthcare. Changing behaviour ingrained in individuals working within health systems will help to ensure the adoption longer term sustainability of the HSS interventions. To address this, we will use the Theoretical Domains Framework (TDF), a determinant framework that brings together evidence based determinants of behaviour.(19) We also use the Behaviour Change Wheel (BCW), that explicitly maps behavioural change interventions onto determinants of behaviour identified with the TDF.(43) Each work-package selects the contextual and behavioural determinants, implementation outcomes and HSS interventions from the different frameworks that are relevant to their aims and objectives. Table 3 describes the objectives of the different implementation science frameworks across the different phases of ASSET. However, identifying determinants that influence the effectiveness of HSS interventions in delivering evidence-informed practices is not enough to address complexity associated with HSS. We will therefore also explore how context influences the mechanisms introduced by the HSS interventions on implementation outcomes.

Table 3: Application of implementation science frameworks across ASSET health system strengthening interventions and research phases

Implementation framework	Pre-implementation phase	Piloting phase	Implementation and Evaluation phase
**CFIR, CICI frameworks will be used to identify determinants of the implementation process including barriers/drivers and contextual influences**	Identify barriers and/or enablers that may influence the delivery of high-quality care;<br><br>Identified barriers are used to select a set of relevant HSS interventions.	Evaluation of selected HSS interventions to understand whether they interact with contextual barrier and/or enabler as intended.	Monitor the influence of identified barriers on the effectiveness of the selected HSS interventions;<br><br>Understand/explain contextual determinants that influence implementation outcomes.
**TDF will be used to identify areas for behavioural change interventions to enhance implementation**	Identify determinants of behaviours that are known to influence healthcare professionals' ability to deliver high quality care;<br><br>Identified determinants are used to select relevant behaviour change interventions.	Evaluation of behaviour change interventions to determine whether they interact with relevant determinant of behaviour as intended.	Understand/explain what behaviours influence implementation outcomes;<br><br>Guide the evaluation of behaviours on implementation outcomes;

For peer review only - http://bmjopen.bmj.com/site/about/guidelines.xhtml

**Behaviour Change Wheel**	Used in combination with the TDF framework to help identify appropriate behavioural change interventions.	Used in combination with the TDF to help identify any additional behavioural change interventions.	NA
**Proctor et al framework, RE-AIM framework to structure the evaluation of the implementation**	Identify implementation outcomes relevant to the aims and objectives of the work package.	Evaluate relevant implementation outcomes including acceptability, appropriateness, feasibility,	Ongoing assessment and adjustment of implementation outcomes at relevant stages of implementation

For peer review only

Measures and taxonomies

Implementation outcomes

To evaluate the effectiveness of the implementation efforts, ASSET uses a combination of implementation outcomes as defined by different evaluation frameworks.(16, 17) The selected implementation outcomes are aligned with the aims and objectives for the different work-packages.

Many measures used to evaluate implementation outcomes have not been validated, making it difficult to compare the effectiveness of alternative interventions both within and between studies.(44) However, three implementation outcomes measures including Acceptability of Intervention Measure (AIM), Intervention Appropriateness Measure (IAM), and Feasibility of Intervention Measure (FIM), have demonstrated promising psychometric properties in high-income settings.(44) Work-packages test the applicability of these measures in low-resourced settings by adapting them to their specific context. Each measure includes four questions measured on a Likert scale (completely disagree, disagree, neither agree nor disagree, agree, completely agree). Questions for each measure will be translated and back translated to ensure linguistic and cross-cultural equivalence. The questions will then be reviewed and cognitive testing with local participants performed prior to the tools being used.

Taxonomies

Initially we use the EPOC taxonomy for HSS interventions to label the different interventions selected by work-packages.(35) If an appropriate intervention is not included in this taxonomy, work-packages use the ERIC taxonomy for implementation strategies to label the intervention.(32) To help alleviate the ambiguity in reporting implementation strategies and HSS interventions, we also report any important differences and similarities between the two taxonomies.

For peer review only - http://bmjopen.bmj.com/site/about/guidelines.xhtml

To provide consistency in labelling of the behavioural change interventions, we use the taxonomy of behavioural change interventions that is also aligned with the TDF and the BCW.(45) However, there are important limitations to applying the TDF and BCW to help select interventions that improve the quality of people-centred care that also address issues such as illness-related stigma. As an example, these behavioural change methods were not specifically designed to address the issues surrounding implementation research in low-resource setting such as stigma, that ASSET is focusing on. As such the behavioural change interventions in this taxonomy may not be appropriate. To address this issue and similar issues with the other taxonomies, where the HSS interventions, quality improvement strategy or behavioural change intervention is not described in any taxonomy, we clearly describe the intervention and highlight the issue with the selected taxonomy not describing our intervention.

Reporting frameworks

To ensure consistent and accurate reporting of implementation studies, work-packages will apply the Standards for Reporting Implementation Studies (StaRI).(46)

Data collection procedures

Data collection in the pre-implementation phase

Workshops

At the end of the pre-implementation phase of ASSET, a two-day workshop is held involving relevant participants from all work-packages (i.e. principal investigators, co-investigators, field staff, and research assistants). The objectives of the workshop are to: (1) standardise the implementation methods being applied across the different work-packages, and; (2) collect data from each work-package on contextual and behavioural determinants associated with the delivery of high-quality evidence-informed care and the associated HSS interventions and behavioural change interventions that were selected to help deliver this care.

For peer review only - http://bmjopen.bmj.com/site/about/guidelines.xhtml

For peer review only

Using data collection tools that identify and describe determinants of the implementation process, participants within the different work-packages apply findings from the formative research to identify and record barriers and/or enablers to the delivery of high-quality care. Participants then use the selected determinants to theorise how potential HSS interventions described in the EPOC taxonomy, can assist in delivering high-quality evidence-informed care relevant to their work package. Similar data collection methods and forms are applied to label and record determinants of behaviours that influence the delivery of evidence-informed care. The identified determinants are then be used to select and record behavioural change interventions.

Interviews & focus groups

The workshop is followed-up with semi-structured virtual interviews involving investigators from the different work-packages, to help understand the 'how and why', which led them to select the HSS interventions and associated strategies to facilitate their implementation (or virtual focus groups if more feasible for country teams). The same interviews/focus groups are also used to review and finalise the findings from the workshop and to understand if there are any gaps in evidence that will require the review any additional documentation. We anticipate including a minimum of two investigators from each ASSET work-package (16 investigators minimum sample) to reach saturation of the thematic areas that emerge from their responses.

Data collection in the piloting and rolling implementation phase

Workshops

Throughout the piloting and rolling implementation phase, workshops are held with each work package team, involving relevant participants (principal and co-investigators, field staff, research assistants, and PhD students). The purpose of the workshops is to guide investigators on how to design the data collection tools including interview guides, using the implementation science determinant

For peer review only - http://bmjopen.bmj.com/site/about/guidelines.xhtml

frameworks, HSS taxonomies and implementation outcomes. In doing so, we hope to ensure findings are standardised and therefore comparable across work-packages.

At the end of the piloting and rolling implementation phase, a separate workshops will be held with each of different work-package teams to feedback the following information collected using the data collection tools: (1) the effectiveness of HSS interventions on standardised implementation outcomes, (2) the influence of context on the effectiveness of HSS intervention in delivering evidence-informed care, and (3) the direct influence of context on the mechanisms introduced by the intervention to produce change.

At the end of the piloting and rolling implementation phase, another workshop lasting one day and involving investigators from all work-packages is used to reflect on the different implementation science methodologies and suggest improvements for further use. Specifically, we seek investigators' opinions about the extent to which different frameworks capture the complexity of how the health system impacts on the overarching problem each work-package is trying to address. Again, we anticipate including a minimum of two investigators from each ASSET work-package (16 investigators minimum sample) to reach saturation of the thematic areas that emerge from their responses. All meetings will be audio-recorded with descriptive notes of discussion. Findings from these workshops are used to suggest how frameworks can be adapted to capture this complexity.

Surveys

In select work packages (WP 1, 2, 5, 6, 8) staff delivering the interventions (i.e. nurses, community health workers) are asked to complete the linguistically adapted tools for the three outcome measures, including acceptability (AIM), appropriateness (IAM) and feasibility (FIM).(44) Approximately 60 staff members from each of the participating work packages contribute to these surveys. A subset of these participants are then interviewed to discuss the usefulness of the data

For peer review only - http://bmjopen.bmj.com/site/about/guidelines.xhtml

collection tools. In particular, we review methods to capture the influence of context on both the effectiveness of the HSS interventions in influencing implementation outcomes as well as the influence of context on influencing the mechanisms introduced by the HSS interventions on implementation outcomes. Participants are also questioned on recommendations to improve our ability to capture the influence of context on implementation outcomes.

Documentary analysis

Theory-of-Change (ToC) methodology is a participatory approach involving key stakeholders that allows the articulation of the 'theory' of how a complex interventional programme will work in reality, describing the necessary interventions to bring about the change, as well as the assumptions inherent to the programme and importantly the context of implementation.(47) ASSET work-packages are developing ToCs to support their implementation and evaluation planning. ToCs are effectively programme theories, contextualised within each one of the ASSET work-packages, offering an overview of how the selected HSS interventions are theorised to achieve specific implementation outcomes. ToCs also include information on key assumptions and work-package context. Each work package including all relevant stakeholders, develops and initial ToC in the pre-implementation phase. This programme theory is then adapted throughout the piloting and pre-implementation phase. We examine whether and how the ToCs align with findings from both the pre-implementation and piloting and rolling implementation phases of the different work-packages including determinants identified using the implementation science frameworks outlined in Table 3. We will also use ToC to better articulate how context influences mechanisms introduced by the HSS interventions described by each work package.

Data Analysis

Psychometric assessment of outcome measures AIM, IAM and FIM

For peer review only - http://bmjopen.bmj.com/site/about/guidelines.xhtml

The outcome measures AIM, IAM and FIM that have demonstrated promising psychometric properties in high-income countries, are tested for similar properties including substantive and discriminant content validity (the extent to which a measure is judged to be reflective of a construct of interest) and inter-item consistency (extent to which scale items are scored in a similar manner) by the different work packages (45) to determine their relevance in low-resource settings. Measures are adapted to the local context, translated and back translated.

To test for substantive validity (extent to which a measure is reflective of the construct of interest) and discriminant content validity (extent to which a measure that are not supposed to be related are actually unrelated,) different cadres of workers who are responsible for delivering the intervention in the different work packages assign 31 items reflecting the three constructs, to each of the three constructs and rate their confidence in the assignments in order test. The Wilcoxon one-sample signed rank test or t-test (as appropriate) is used to determine whether items measured the intended construct, or whether items measured a combination of constructs. Hochberg's correction is used to correct for multiple tests.(48) Intraclass correlation coefficients (ICCs) using a two-way mixed effects model to assess the level of agreement in item assignments among all participants, and also within key stakeholder groups, across 31 items and for each construct.(49) The same data are also used to assess the factorial validity of the three measures, initially through exploratory factor analyses.(44) We assess inter-item consistency by computing Cronbach alpha for each of the four-item scales. For each measure, we also calculate means and standard deviations. Higher scores represent more favourable responses. If the measures demonstrate adequate psychometric properties, they are used to facilitate cross-site comparisons across the different work-packages.

Data collected by ASSET-ImplementER

For peer review only - http://bmjopen.bmj.com/site/about/guidelines.xhtml

For each work package, a thematic analysis is used to analyse the qualitative data collected in semi-structured interviews and workshops that identify key issues pertinent to using implementation science frameworks and implementation outcome measures for HSS in low-resourced contexts.

Analysis and synthesis of data collected by different work-packages

At the end of each phase of research, data is collected from each of the work packages (i.e. contextual and behavioural determinants, mechanisms, selected HSS interventions and implementation outcomes) and entered into a template created in Excel software. As an example, each work-package records information on identified barriers/enablers, associated data source, relevant implementation science framework, EPOC HSS interventions, and implementation outcomes. Table 4 demonstrates an example of the Excel template.

Table 4: Sample template used to synthesise findings from the workshops and interviews

Determinant	Data source for determinants	Implementation science framework	EPOC health system strengthening intervention	ERIC implementation strategy	Behavioural change intervention
Illness-related stigma	Focus-group discussion with key stakeholders	CICI/TDF	Educational meetings with health care providers, educational materials distributed to patients and clinicians	Education and training	Motivate health workers

We adapt the matrixed multiple case study approach to analyse and synthesise the data we record in the templates.(50) This method facilitates comparisons between relevant work-packages, organising, analysing and presenting common and heterogeneous findings across implementation sites. Such an approach also aims to create generalisable knowledge regarding what and how local factors influence implementation.The matrixed multiple case study approach uses a combination of quantitative and qualitative methods that will allow us to identify associations between specific implementation processes and contextual factors on the one hand, and implementation outcomes on the other.

Initially, data are analysed separately for each work package. Quantitative data are analysed using means and proportions. Given the heterogeneous nature of the care platforms and associated work-

For peer review only - http://bmjopen.bmj.com/site/about/guidelines.xhtml

packages, context, and selected HSS interventions, it will not be useful to compare quantitatively the influence of context on the effectiveness of HSS interventions on implementation outcomes between the work-packages. Instead, we use a qualitative approach that aims to understand why implementation outcomes were similar or different by describing the associated HSS interventions and contextual and behavioural determinants.

Results from the matrixed multiple case study approach as well as findings from the interviews and the final workshop will be triangulated to identify points of convergence and divergence between different work-packages. Using these methods, we analyse how each work-package defined and operationalised different implementation science constructs (e.g. context, intervention mechanisms, HSS interventions, behaviour change techniques), and how implementation theories and frameworks were used to support specific HSS interventions throughout implementation. The final part of our analysis integrates these elements to offer an overarching understanding of how implementation science frameworks have been operationalised across all ASSET work-packages and whether the use of these frameworks offered added utility (from a design, implementation or evaluation perspectives).

For peer review

Patient public involvement

Patients and the public were not involved in the designing/writing protocol for this protocol. However, extensive participatory methods that involve both the patients and public will be used by the individual work packages to design, select and evaluate the HSSIs for ASSET.

Ethics

The ASSET-ImplementER theme is considered minimal risk as we only interview researchers involved in the different work packages. To this effect we have received approval from King's College London

For peer review only - http://bmjopen.bmj.com/site/about/guidelines.xhtml

Ethics Committee for research that is considered minimal risk (Reference number: MRA-20/21-21772).

Discussion

To our knowledge, ASSET-ImplementER is one of the first global health implementation research programmes that attempts to standardise methodologies to design and evaluate HSS interventions across different healthcare platforms and settings. In doing so, we expect to improve our understanding of what HSS interventions work for whom, by creating a compilation of HSS interventions used by the different work-packages, the associated barriers, and the effectiveness on implementation outcomes.

ASSET-ImplementER will also attempt to develop novel insights into how we can improve implementation science methodologies for designing implementation research on HSS in LMICs. Importantly, there is a lack of standardisation in not only the terminology used to describe implementation research and HSS, but also the methods applied to this research. By labelling contextual and behavioural determinants using established frameworks and testing psychometric properties of implementation outcome measures that have demonstrated similar properties in high-income countries, we hope to improve our ability to compare the effectiveness of different HSS interventions. Importantly, many researchers find the plethora of implementation science frameworks and theories overwhelming and difficult to apply in practice. Through interviews with different stakeholders, we hope to improve methods for HSS in LMICs including our ability to learn how to best apply and simplify frameworks across different health systems.

There are limitations to ASSET-ImplementER. Although our programme is theory-informed through the use of ToC workshops and implementation science determinant frameworks, we have not used implementation science theories or other middle-range theories to guide the design of ASSET as a

For peer review only - http://bmjopen.bmj.com/site/about/guidelines.xhtml

programme. Indeed, many people criticise determinant frameworks (which we apply heavily within ASSET) as being `a' theoretical. Nevertheless, given many of the work-packages teams were unfamiliar with implementation science methods at the time of ASSET set-up, we feel this is a pragmatic approach to applying a theory-informed approach to HSS. To mitigate the effects of this static approach to our research, we work with the different work packages throughout the different phases of research, to theorise and conceptualise how the selected determinants interact with mechanisms introduced by the selected HSS on implementation outcomes.

ASSET-ImplementER applies a system-level approach to both the design and evaluation of HSS interventions for the ASSET programme. Although there are limitations to our approach, we expect to begin advance our understanding of what HSS work for whom, and how. It is also hoped that we will start to address the issues in understanding the complexity surrounding how to effectively strengthen health systems in resource-poor contexts within LMICs.

Figure 1: Flow of methods for the ASSET-ImplementER theme

For peer review only

References

1. Sachs J. From Millennium Development Goals to Sustainable Development Goals. The Lancet. 2012;379(9832):2206-11.
2. United Nations. Transforming our world: the 2030 Agenda for Sustainable Development http://www.un.org/ga/search/view_doc.asp?symbol=A/RES/70/1&Lang=E. 2015.
3. Bickler SN WT, Kassebaum N,. In: Debas HT, Donkor P, Gawande A, et al., editors. Essential Surgery: Disease Control Priorities, Third Edition (Volume 1). Washington (DC): The International Bank for Reconstruction and Development / The World Bank; 2015 Apr 2. Chapter 2. Available from: https://www.ncbi.nlm.nih.gov/books/NBK333518/ doi: 10.1596/978-1-4648-0346-8_ch22015.
4. World Health Organization. Everybody business : strengthening health systems to improve health outcomes : WHO's framework for action. Geneva, Switzerland; 2007.
5. Witter S, Palmer N, Balabanova D, Mounier-Jack S, Martineau T, Klicpera A, et al. Health system strengthening—Reflections on its meaning, assessment, and our state of knowledge. The International journal of health planning and management. 2019;34(4):e1980-e9.
6. De Savigny D, Adam T. Systems thinking for health systems strengthening. Alliance for Health Policy
and Systems Research,WHO. 2009.
7. Chee G, Pielemeier N, Lion A, Connor C. Why differentiating between health system support and health system strengthening is needed. The International journal of health planning and management. 2013;28(1):85-94.
8. World Health Organization. Everybody business: strengthening health systems to improve health outcomes: WHO's framework for action. Geneva. WHO. 2007.
9. Borghi J, Chalabi Z. Square peg in a round hole: re-thinking our approach to evaluating health system strengthening in low-income and middle-income countries. BMJ Global Health. 2017;2(3):e000406.
10. Peters DH, Adam T, Alonge O, Agyepong IA, Tran N. Implementation research: what it is and how to do it. BMJ. 2013;347.
11. Peters D TN, Adam T,. Implementation research in health: a practical guide. Alliance for Health Policy and Systems Research, World Health Organization, 2013.
12. Theobald S, Brandes N, Gyapong M, El-Saharty S, Proctor E, Diaz T, et al. Implementation research: new imperatives and opportunities in global health. The Lancet. 2018;392(10160):2214-28.
13. Eccles M, Mittman B. Welcome to Implementation Science. Implementation Science. 2006;1(1):1.
14. Tabak R, Khoong E, Chambers D, Brownson R. Bridging research and practice: models for dissemination and implementation research. Am J Prev Med. 2012;43(3):337-50.
15. Nilsen P. Making sense of implementation theories, models and frameworks. Implementation Science. 2015;10(1):53.
16. Proctor E, Silmere H, Raghavan R, Hovmand P, Aarons G, Bunger A, et al. Outcomes for Implementation Research: Conceptual Distinctions, Measurement Challenges, and Research Agenda. Administration and policy in mental health. 2011;38(2):65-76.

For peer review only - http://bmjopen.bmj.com/site/about/guidelines.xhtml

17. Glasgow RE, McKay HG, Piette JD, Reynolds KD. The RE-AIM framework for evaluating interventions: what can it tell us about approaches to chronic illness management? Patient Educ Couns. 2001;44.
18. Craig P, Di Ruggiero E, Frohlich KL. Chapter 3, Taking account of context in the population health intervention research process. Canadian Institutes of Health Research (CIHR) - National Institute for Health Research (NIHR). Southampton (UK): NIHR Journals Libary; 2018.
19. Atkins L, Francis J, Islam R, O'Connor D, Patey A, Ivers N, et al. A guide to using the Theoretical Domains Framework of behaviour change to investigate implementation problems. Implementation Science. 2017;12(1):77.
20. Rogers PJ. Using Programme Theory to Evaluate Complicated and Complex Aspects of Interventions. Evaluation. 2008;14(1):29-48.
21. Adam T, Hsu J, de Savigny D, Lavis J, Rottingen J, Bennett S. Evaluating health systems strengthening interventions in low-income and middle-income countries: are we asking the right questions? Health Policy Plan. 2012;27 Suppl 4:iv9-19.
22. Alonge O, Rodriguez D, Brandes N, Geng E, Reveiz L, Peters D. How is implementation research applied to advance health in low-income and middle-income countries? BMJ Global Health. 2019;4(2):e001257.
23. Ridde V. Need for more and better implementation science in global health. BMJ Global Health. 2016;1(2):e000115.
24. Ridde V, Pérez D, Robert E. Using implementation science theories and frameworks in global health. BMJ Global Health. 2020;5(4):e002269.
25. Pantoja T, Opiyo N, Lewin S, Paulsen E, Ciapponi A, Wiysonge CS, et al. Implementation strategies for health systems in low-income countries: an overview of systematic reviews. The Cochrane database of systematic reviews. 2017;9(9):CD011086-CD.
26. Colquhoun H, Leeman J, Michie S, Lokker C, Bragge P, Hempel S, et al. Towards a common terminology: a simplified framework of interventions to promote and integrate evidence into health practices, systems, and policies. Implementation Science. 2014;9(1):781.
27. McKibbon K, Lokker C, Wilczynski N, Ciliska D, Dobbins M, Davis DA, et al. A cross-sectional study of the number and frequency of terms used to refer to knowledge translation in a body of health literature in 2006: a Tower of Babel? Implementation Science. 2010;5(1):16.
28. Eccles MP, Mittman BS. Welcome to Implementation Science. Implementation Science. 2006;1(1):1.
29. Shojania K, Grimshaw J. Evidence-Based Quality Improvement: The State Of The Science. Health Affairs. 2005;24(1):138-50.
30. Cotterill S, Knowles S, Martindale AM, Elvey R, Howard S, Coupe N, et al. Getting messier with TIDieR: embracing context and complexity in intervention reporting. BMC Medical Research Methodology. 2018;18(1):12.
31. Hoffmann T, Glasziou P, Boutron I, Milne R, Perera R, Moher D, et al. Better reporting of interventions: template for intervention description and replication (TIDieR) checklist and guide. BMJ : British Medical Journal. 2014;348:g1687.
32. Powell B, Waltz Thomas, Chinman Matthew, Damschroder Laura, Smith Jeffrey, Matthieu Monica, et al. A refined compilation of implementation strategies: results from the Expert Recommendations for Implementing Change (ERIC) project. 2015;10(1):21.

For peer review only

33. Booth A, Moore G, Flemming K, Garside R, Rollins N, Tunçalp Ö, et al. Taking account of context in systematic reviews and guidelines considering a complexity perspective. BMJ Global Health. 2019;4(Suppl 1):e000840.
34. Shojania KG MK, Wachter RM,. Closing the Quality Gap: A Critical Analysis of Quality Improvement Strategies (Vol. 1: Series Overview and Methodology). Rockville (MD): Agency for Healthcare Research and Quality (US); 2004 Aug. (Technical Reviews, No. 9.1.) Available from: https://www.ncbi.nlm.nih.gov/books/NBK43908/
. 2004.
35. Effective Practice and Organisation of Care (EPOC). The EPOC taxonomy of health systems interventions. EPOC Resources for review authors. . Oslo: Norwegian Knowledge Centre for the Health Services; 2016.
36. ASSET Health System StrEngThening in Sub Saharan Africa 2020 [The web page for the NIHR funded ASSET programme].
37. Curran G, Bauer M, Mittman B, Pyne J, Stetler C. Effectiveness-implementation hybrid designs: combining elements of clinical effectiveness and implementation research to enhance public health impact. Medical care. 2012;50(3):217-26.
38. Damschroder L, Aron D, Keith R, Kirsh S, Alexander J, Lowery J. Fostering implementation of health services research findings into practice: a consolidated framework for advancing implementation science. Implementation Science. 2009;4(1):50.
39. Pfadenhauer L, Gerhardus A, Mozygemba K, Lysdahl K, Booth A, Hofmann B, et al. Making sense of complexity in context and implementation: the Context and Implementation of Complex Interventions (CICI) framework. Implementation Science. 2017;12(1):21.
40. Grol R, Grimshaw J. From best evidence to best practice: effective implementation of change in patients' care. Lancet. 2003;362(9391):1225-30.
41. Noar S, Zimmerman R. Health Behavior Theory and cumulative knowledge regarding health behaviors: are we moving in the right direction? Health Education Research. 2005;20(3):275-90.
42. Proctor E, Silmere H, Raghavan R, Hovmand P, Aarons G, Bunger A, et al. Outcomes for implementation research: conceptual distinctions, measurement challenges, and research agenda. Administration and policy in mental health. 2011;38(2):65-76.
43. Michie S, van Stralen M, West R. The behaviour change wheel: A new method for characterising and designing behaviour change interventions. Implementation Science. 2011;6(1):42.
44. Weiner B, Lewis C, Stanick C, Powell BJ, Dorsey CN, Clary AS, et al. Psychometric assessment of three newly developed implementation outcome measures. Implementation science : IS. 2017;12(1):108-.
45. Michie S, Richardson M, Johnston M, Abraham C, Francis J, Hardeman W, et al. The behavior change technique taxonomy (v1) of 93 hierarchically clustered techniques: building an international consensus for the reporting of behavior change interventions. Annals of behavioral medicine : a publication of the Society of Behavioral Medicine. 2013;46(1):81-95.
46. Pinnock H, Epiphaniou E, Sheikh A, Griffiths C, Eldridge S, Craig P, et al. Developing standards for reporting implementation studies of complex interventions (StaRI): a systematic review and e-Delphi. Implementation Science. 2015;10(1):42.
47. Vogel I. Review of the use of theory of change in international development. UK: Department for International Development, 2012.

For peer review only - http://bmjopen.bmj.com/site/about/guidelines.xhtml

48. Hochberg Yosef. A sharper Bonferroni procedure for multiple tests of significance. Biometrika. 1988;75(4):800-2.
49. Dixon D, Pollard B, Johnston M. What does the chronic pain grade questionnaire measure? Pain. 2007;130(3):249-53.
50. Kim B, Sullivan J, Ritchie M, Connolly SL, Drummond KL, Miller CJ, et al. Comparing variations in implementation processes and influences across multiple sites: What works, for whom, and how? Psychiatry Research. 2019:112520.

Declarations

Competing interests: None declared

Patient consent: Protocol paper so consent not applicable

Data sharing statement: No additional data available as a protocol paper

Ethics: Approval from King's College London Ethics Committee for research that is considered minimal risk (Reference number: MRA-20/21-21772).

Funding: The study is funded by the National Institute of Health Research (NIHR) Global Health Research Unit on Health System Strengthening in Sub-Saharan Africa, King's College London (GHRU 16/136/54) using UK aid from the UK Government to support global health research. GT, WG and NS' research is also supported by the National Institute for Health Research (NIHR) Applied Research Collaboration (ARC) South London at King's College Hospital NHS Foundation Trust. GT and NS are members of King's Improvement Science, which offers co-funding to the NIHR ARC South London and is funded by King's Health Partners (Guy's and St Thomas' NHS Foundation Trust, King's College Hospital NHS Foundation Trust, King's College London and South London and Maudsley NHS Foundation Trust), Guy's and St Thomas' Charity and the Maudsley Charity. NS' research is further supported by the ASPIRES research programme (Antibiotic use across Surgical Pathways - Investigating, Redesigning and Evaluating Systems), funded by the Economic and Social Research Council. GT also receives support from the National Institute of Mental Health of the National Institutes of Health under award number R01MH100470 (Cobalt study) and by the UK Medical Research Council in relation the Emilia (MR/S001255/1) and Indigo Partnership (MR/R023697/1) awards. The views expressed are those of the authors and not necessarily those of the NHS, the NIHR,

For peer review only

the ESRC, the MRC, the charities or the Department of Health and Social Care. CH receives support from AMARI as part of the DELTAS Africa Initiative (DEL-15-01).

Authors contributions:

NSeward wrote the first and subsequent drafts of the paper

NSeward and NSevdalis conceptualised the idea for the paper

NSeward, NSevdalis, Jamie Murdoch and Charlotte Hanlon offered insight and edited all drafts of the paper

All other authors (RA, WG, RH, CL, SHK, RM, MK, MP, JS, AS, GT, RV) edited and offered input to various drafts of the paper

For peer review only

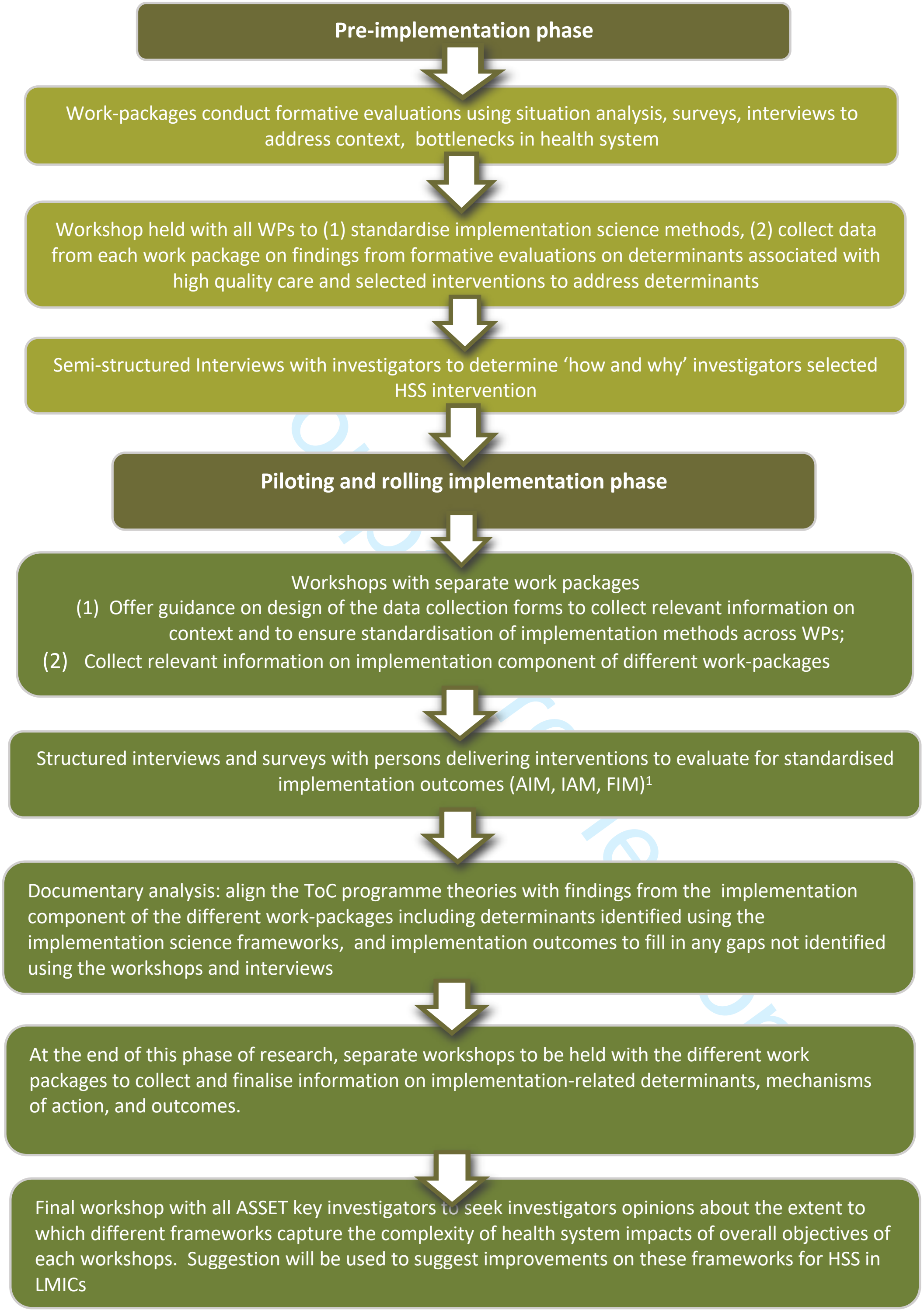

1. Acceptability of Intervention Measure (AIM), Intervention Appropriateness Measure (IAM), and Feasibility of Intervention Measure (FIM), have demonstrated promising psychometric properties in high-income settings.(38)

Figure 1: Flow of methods for the ASSET-ImplementER theme

BMJ Open

Implementation Science Protocol for a participatory, theory-informed implementation research programme in the context of health system strengthening in sub-Saharan Africa (ASSET-ImplementER)

Journal:	*BMJ Open*
Manuscript ID	bmjopen-2021-048742.R1
Article Type:	Protocol
Date Submitted by the Author:	01-Jun-2021
Complete List of Authors:	Seward, Nadine; King's College London, Centre for Implementation Science, Department of Health Services and Population Research<br>Murdoch, Jamie; University of East Anglia, School of Health Sciences<br>Hanlon, Charlotte; King's College London, Institute of Psychiatry, Psychology and Neuroscience, Health Service and Population Research Department, Centre for Global Mental Health; Addis Ababa University College of Health Sciences, School of Medicine, Department of Psychiatry, WHO Collaborating Centre for Mental Health Research and Capacity-Building<br>Araya, Ricardo; Centre for Global Mental Health and Primary Care Research,<br>Gao, Wei; King's College London, Palliative Care, Policy & Rehabilitation<br>Harding, Richard; King's College London, Dept of Palliative Care and Policy<br>Lund, Crick; King's College London, Centre for Global Mental Health, Department of Health Service and Population Research Department; University of Cape Town, Alan J Flisher Centre for Public Mental Health, Department of Psychiatry and Mental Health<br>Hinrichs-Krapels, Saba; King's College London, The Policy Institute; King's College London, King's Global Health Institute<br>Mayston, Rosie; King's College London, King's Global Health Institute; King's College London, Global Health and Social Medicine<br>Kartha, Muralikrishnan ; King's College London, King's Health Economics<br>Prince, Martin; King's College London, King's Global Health Institute<br>Sandall, Jane; King's College London, Women and Children's Health, School of Life Course Sciences<br>Thornicroft, Graham; King's College London, Centre for Implementation Science, Department of Health Services and Population Research; King's College London, Centre for Implementation Science, Health Services and Population Research<br>Verhey, Ruth ; Friendship Bench Zimbabwe,<br>Sevdalis, Nick; King's College London, Centre for Implementation Science, Department of Health Services and Population Research
**Primary Subject Heading**:	Global health
Secondary Subject Heading:	Health services research, Public health

Keywords:	PUBLIC HEALTH, STATISTICS & RESEARCH METHODS, TROPICAL MEDICINE

SCHOLARONE™ Manuscripts

I, the Submitting Author has the right to grant and does grant on behalf of all authors of the Work (as defined in the below author licence), an exclusive licence and/or a non-exclusive licence for contributions from authors who are: i) UK Crown employees; ii) where BMJ has agreed a CC-BY licence shall apply, and/or iii) in accordance with the terms applicable for US Federal Government officers or employees acting as part of their official duties; on a worldwide, perpetual, irrevocable, royalty-free basis to BMJ Publishing Group Ltd ("BMJ") its licensees and where the relevant Journal is co-owned by BMJ to the co-owners of the Journal, to publish the Work in this journal and any other BMJ products and to exploit all rights, as set out in our licence.

The Submitting Author accepts and understands that any supply made under these terms is made by BMJ to the Submitting Author unless you are acting as an employee on behalf of your employer or a postgraduate student of an affiliated institution which is paying any applicable article publishing charge ("APC") for Open Access articles. Where the Submitting Author wishes to make the Work available on an Open Access basis (and intends to pay the relevant APC), the terms of reuse of such Open Access shall be governed by a Creative Commons licence – details of these licences and which Creative Commons licence will apply to this Work are set out in our licence referred to above.

Other than as permitted in any relevant BMJ Author's Self Archiving Policies, I confirm this Work has not been accepted for publication elsewhere, is not being considered for publication elsewhere and does not duplicate material already published. I confirm all authors consent to publication of this Work and authorise the granting of this licence.

For peer review only

Implementation Science Protocol for a participatory, theory-informed implementation research programme in the context of health system strengthening in sub-Saharan Africa (ASSET-ImplementER)

Nadine Seward[1], Jamie Murdoch[2], Charlotte Hanlon [3,4,] Ricardo Araya[4], Wei Gao[5], Richard Harding[5], Crick Lund[4,6], Saba Hinrichs-Krapels[7,8], Rosie Mayston[8], Muralikrishnan R. Kartha[9], Martin Prince[8], Jane Sandall[10], Graham Thornicroft[1,4], Ruth Verhey[11], Nick Sevdalis[1]

Corresponding author: Dr Nadine Seward (nadine.seward@kcl.ac.uk) , 16 De Crespigny Park, Camberwell, London SE5 8AB, nadine.seward@kcl.ac.uk

Affiliations:

1. Centre for Implementation Science, Health Service and Population Research Department, Institute of Psychiatry, Psychology & Neuroscience, King's College, UK
2. School of Health Sciences, University of East Anglia
3. Department of Psychiatry, WHO Collaborating Centre for Mental Health Research and Capacity-Building, School of Medicine, College of Health Sciences, Addis Ababa University, Ethiopia
4. Centre for Global Mental Health, Health Service and Population Research Department, Institute of Psychiatry, Psychology & Neuroscience, King's College London, UK
5. Florence Nightingale Faculty of Nursing Midwifery and Palliative Care, Cicely Saunders Institute
6. Alan J Flisher Centre for Public Mental Health, Department of Psychiatry and Mental Health, University of Cape Town
7. The Policy Institute, King's College London
8. King's Global Health Institute, King's College London, UK
9. King's Health Economics, Kings College London
10. Dept of Women and Children's Health, School of Life Course Sciences, FoLSM, Kings College London
11. Research Support Centre, College of Health Sciences, University of Zimbabwe

For peer review only

Abbreviations

For peer review only - http://bmjopen.bmj.com/site/about/guidelines.xhtml

ASSET: Global Research Unit on Health System Strengthening in sub-Saharan Africa
AIM: Acceptability of Intervention Measure
ASSET-ImplementER: Implementation science theme within ASSET
BCW: Behavioural Change Wheel
CFIR framework: Consolidated Framework for Implementation Research
CICI framework: Context and Implementation of Complex Interventions
EPOC taxonomy: Effective Practice and Organisation of Care taxonomy for health system strengthening interventions
ERIC taxonomy: Expert Recommendations for Implementing Change taxonomy of implementation strategies
FIM: Feasibility of Intervention Measure
HSS: Health systems strengthening
IAM: Intervention appropriateness Measure
LMICs: Low- and Middle-Income Countries
NIHR: National Institute of Health Research
STaRI: Standards for Reporting Implementation Studies
TDF: Theoretical Domains Framework
ToC: Theory of Change
WP: Work package

Word count: 5399

Key words: implementation research, implementation science, health systems strengthening, global health, Sub-Saharan Africa

For peer review only

ABSTRACT

Objectives

ASSET (Health System Strengthening in Sub-Saharan Africa) is a health system strengthening (HSS) programme involving eight work-packages (i.e. a research study that addresses a specific need for HSS) that aims to develop solutions that support high-quality care. Here we present the protocol for the implementation science (IS) theme within ASSET (ASSET-ImplmentER) that aims to understand what HSS interventions work, for whom and how, and how implementation science methodologies can be adapted to improve the HSS interventions within resource-poor contexts.

Settings

Publicly funded health facilities in rural and urban areas in in Ethiopia, South Africa, Sierra Leone, and Zimbabwe.

Participants

Research staff including principal investigators, co-investigators, field staff, PhD students, and research assistants.

Interventions

Work-packages use a mixed-methods effectiveness-effectiveness hybrid designs. At the end of the pre-implementation phase, a workshop is held whereby the IS theme, jointly with ASSET work-packages apply IS determinant frameworks to research findings to identify factors that influence the effectiveness of delivering evidence-informed care. Determinants are used to select a set of HSS interventions for further evaluation, where work-packages also theorise selective mechanisms.

In the piloting and rolling implementation phase, work-packages pilot the HSS interventions. An iterative process then begins involving evaluation, reflection and adaptation. Throughout this phase, IS determinant frameworks are applied to monitor and identify barriers/enablers to implementation.

For peer review only - http://bmjopen.bmj.com/site/about/guidelines.xhtml

Selective mechanisms of action are also investigated. Implementation outcomes are evaluated using qualitative and quantitative methods. The psychometric properties of outcome measures including acceptability, (appropriateness, and feasibility are also evaluated. In a final workshop, work-packages come together, to reflect and explore the utility of the selected IS methods and provide suggestions for future use.

Structured templates are used to organise and analyse common and heterogeneous patterns across work-packages. Qualitative data are analysed using thematic analysis and quantitative data is analysed using means and proportions.

Conclusions

We use a novel combination of IS methods at a programmatic level to facilitate comparisons of determinants and mechanisms that influence the effectiveness of HSS interventions in achieving implementation outcomes across different contexts. The study also contributes conceptual development and clarification at the underdeveloped interface of implementation science, HSS and global health.

Strengths and limitations of this study

- *ASSET-ImplementER aims to advance understanding of how to use implementation science to design and evaluate HSS interventions across different health systems and contexts.*
- *Implementation science determinant and evaluation frameworks were selected to help conceptualise what health systems strengthening interventions work for whom and how.*
- *Research staff working within the different work-packages are using determinant frameworks to inform their theorising of how proposed determinants interact to produce mechanisms and the identified outcomes.*
- *Work-packages will also validate measures of implementation outcomes in LMICs including acceptability (AIM), appropriateness (IAM) and feasibility (FIM).*
- *The research will provide recommendations for harnessing implementation science to effectively strengthen health systems in resource-poor contexts in LMICs.*

For peer review only - http://bmjopen.bmj.com/site/about/guidelines.xhtml

BACKGROUND

The United Nations Third Sustainable Development Goal (SDG3) includes a call for Universal Health Coverage (UHC), particularly within research poor contexts within LMICs.(1) Achieving UHC that explicitly addresses the availability and delivery of high-quality evidence-informed care has been identified as an urgent priority for health system strengthening in low- and middle-income countries (LMICs).(2, 3)

The scale of unmet need is reflected in estimates that that suggest 5 million people in LMICs who engaged with the health system died due to poor-quality health care in 2016, and that 3.6 million deaths occurred due to people not accessing care at all.(4) Simply increasing access to care, does not necessarily improve quality of care and improve health outcomes.(5) A stark example of this issue is evident with the Janani Suraksha Yojana (JSY), a cash incentives programme targeting women who lived below the poverty line in India, to deliver in a health facility.(6, 7) Despite the increased coverage of facility-based deliveries, there was no corresponding improvement with maternal and newborn outcomes. Improving population health will require not only increased access to health services, but also the provision of high-quality care.(8)

There is lack of consensus as to what constitutes health systems strengthening (HSS).(9) Initially, the term HSS was a reaction to the limitations of vertical programmes targeting specific diseases, that do not strengthen the broader health system.(9) Over time, the definition of HSS changed to one that has system-level effects and not just organisational level effects. It is now recognised that health systems are dynamic and complex socio-technical structures, composed of multiple interacting components that are constantly adapting to changes in the local context and therefore behave unpredictably.(10) HSS to improve quality of care is about "*permanently making the system function better, and not just about filling gaps or supporting the system to produce better short-term outcomes*".(11) HSS involves

For peer review only - http://bmjopen.bmj.com/site/about/guidelines.xhtml

comprehensive changes to policies and regulations, organisational structures, and relationships across the health system building blocks (e. g. service delivery, health workforce, health information systems, access to essential medicines, financing, and leadership/governance) that motivate changes in behaviour, and/or allow more effective use of resources across multiple care platforms.(10, 12)

Interventions to strengthen health systems, by their very nature, improve health outcomes by providing strategies that influence several mechanisms, both simultaneously and in isolation, at various time points and at various levels of the health system.(10) As such, HSS requires an approach to design and evaluate complex public health interventions, in real-world contexts, that accounts for the multiple interconnecting components and actors.(13) A systems-level approach to the design and evaluation of HSS interventions views a complex intervention as a system in itself, interacting with other building blocks of the underlying health system in which the intervention embeds itself, setting off reactions that may well be unexpected or unpredictable.(10) Applying this approach to the design and evaluation of HSS interventions requires an evaluation of not only their main effects, but also inputs, outputs, initial, intermediate and eventual outcomes, feedback processes and contexts within the underlying health system.(10)

Applying implementation science concepts and methods to health system strengthening

Implementation research is a rapidly expanding discipline that seeks to understand what, why, and how interventions work in real-world settings.(14) Implementation research can consider any aspect of implementation, including understanding contextual and behavioural barriers that influence implementation efforts, the process of implementation, and the evaluation of implementation efforts for outcomes such as reach, fidelity and sustainability.(14, 15) By addressing implementation challenges through the participation of actors from multiple disciplines, implementation research can help to apply a systems-level approach and extract lessons that contribute to building stronger and more resilient and responsive health systems.(16)

For peer review only - http://bmjopen.bmj.com/site/about/guidelines.xhtml

Whereas implementation *research* seeks to understand what, why, and how interventions work in real-world settings,(14) implementation science is the study of methods used to carry out this research.(17) Specifically, implementation science offers theories, models, frameworks and other methodologies to optimise and evaluate the implementation of evidence-informed care.(17) The methods offered through implementation science can inform decision-makers on how best to select and adapt HSS interventions to implement the evidence-informed care within the dynamic environments in which they work.(18) For example, implementation science determinant frameworks describe contextual and behavioural barriers that are known to influence implementation outcomes, and include key factors to consider in the evaluation of the process of implementation.(19) Health system strengthening interventions are selected to address specific barriers to implement the evidence-informed care.

ASSET (Health System Strengthening in Sub-Saharan Africa) is a health system strengthening (HSS) programme that aims to develop and solutions that support high-quality care. The implementation science (IS) theme within ASSET (ASSET-ImplmentER) aims to ensure rigorous and appropriate methodologies are applied across ASSET. Box 1 describes key implementation science definitions as used within the ASSET-ImplementER theme.

Box 1: Implementation science terminology used within the ASSET-ImplementER programme

Implementation strategies: methods or techniques used to enhance the adoption, implementation and sustainability of a clinical program or practice. Other terminology includes HSS interventions, quality improvement strategies.

Implementation outcomes: Implementation outcomes assess how well implementation has occurred or provide insights about how this contributes to an individual's health status or other important health outcomes. Proctor et al articulate the following core set of eight implementation outcomes: acceptability, feasibility, uptake, penetration, cost, fidelity, and sustainability.(20)

Implementation effectiveness: the impact of implementation efforts on implementation outcomes.

For peer review only - http://bmjopen.bmj.com/site/about/guidelines.xhtml

Evaluation framework: Specifies implementation outcomes that can be evaluated to determine implementation effectiveness.(21)

Context: Any feature of the circumstances in which an intervention is conceived, developed, implemented and evaluated.(22) Contextual features are intervention specific but include: (1) whole population and compositional features (varying between individuals within a population); (2) features of the physical location or geographical setting of interventions, as well as cultural, social, economic and political aspects; and (3) features affecting implementation (organisation, funding, policy, etc.), as well as those directly affecting outcomes.

Determinant frameworks: identify contextual barriers and/or enablers that are known to impact on the effectiveness of implementation efforts.(19)

Theoretical frameworks: identify determinants of behaviours that are known to influence implementation outcomes.(23)

Implementation theories/middle range theories: describe the mechanisms behind how a proposed intervention works. These theories can also be used to identify barriers and/or enablers to change and what needs to change.(19)

Programme theory: describes how a specific intervention is expected to lead to its effects and under what conditions.(24)

Need for improved methods to design and evaluate health system strengthening interventions in LMICs

Despite the need to account for the complexity of HSS interventions to implement evidence-informed care, current methods used to design and evaluate such interventions in LMICs typically lack robust methodologies that enable an understanding of what interventions worked, for whom, and how.(25) As an example, few studies to-date have been supported by a conceptual framework (including a programme theory) explaining how an intervention is theorised to work, or a detailed process evaluation that helps to explain whether the intervention worked as intended, and if so, for whom, and under what circumstances.(25-28) There are also limitations with how studies report how the local context influences the effectiveness of HSS interventions on important implementation outcomes such as coverage, acceptability and fidelity. Importantly, much of the evidence base for HSS interventions still comes from high-income countries, with uncertain generalisability of evidence to low-resourced settings.(29)

Compounding the methodological shortcoming with HSS in LMICs, are the inconsistencies in the terminology used to describe the science of promoting and supporting the use of evidence in health and healthcare policy(30, 31) and also its reporting. As an example, implementation science has been described variously as quality improvement, knowledge translation, knowledge transfer, and even complex intervention evaluation.(31) Although there are differences between fields such

For peer review only - http://bmjopen.bmj.com/site/about/guidelines.xhtml

implementation science and improvement science, they share the objective of improving the quality of healthcare delivery and therefore clinical outcomes.(32, 33)

Perhaps even more important is the lack of consistency in reporting and describing the implementation strategies to improve the quality of service delivery and therefore clinical outcomes.(34, 35) This results in the inability to replicate or generalise research studies to different contexts, or to allow for research synthesis such as meta-analysis or systematic reviews.(36, 37) To help address this issue, the Expert Recommendations for Implementing Change (ERIC) study created a taxonomy of implementation strategies(36) – to allow researchers to apply a common language when describing how evidenced interventions are being implemented. Taxonomies for quality improvement strategies have also been created that are conceptually similar (in some cases identical) to the ERIC taxonomy.(36, 38) As an example, both taxonomies include audit and feedback strategies, provider education, patient education, patient reminders and organisational change.(36, 38) There is also a taxonomy for HSS interventions that includes implementation strategies, the Effective Practice and Organisation of Care (EPOC) taxonomy, that is also similar to the taxonomies for implementation and quality improvement strategies.(36, 39)

There is, therefore, an urgent need to improve not only the quality of implementation research for HSS in LMICs, but also consistency in reporting the implementation strategies used to deliver evidence-informed care and improve service delivery and patient outcomes. For purposes of the ASSET-ImplementER theme, we will refer to these interventions as HSS interventions. Embedding high-quality implementation science methodology in HSS research that includes consistent reporting of the HSS interventions used to deliver evidence-informed care, will help unpack the 'black box' of how such interventions work (or fail to reach their expected potential) for certain populations in a given setting.(26) The ASSET-ImplementER study aims to start addressing some of these issues.

For peer review only - http://bmjopen.bmj.com/site/about/guidelines.xhtml

The National Institute of Health Research (NIHR) Global Research Unit on Health System Strengthening in Sub-Saharan Africa (ASSET) and the ASSET-Implementation Research theme (ASSET-ImplementER)

ASSET is a four-year HSS research programme with a ten month no-cost extension (2017-22) that addresses the imperative of using implementation research with robust implementation science methods that use consistent reporting of the HSS interventions to deliver high-quality care. ASSET spans three healthcare platforms (primary health care for the integrated treatment of chronic conditions in adults, maternal and newborn, surgical care) involving eight work-packages within four different countries within sub-Saharan Africa. A work-package can be defined as a research study that aims to address an identified need for HSS within a care platform. The overall aim of ASSET is to develop, implement and evaluate high-quality HSS interventions that are effective and sustainable. ASSET also aims to ensure care is equitable, people-centred and respectful.(40) ASSET is one of the first implementation research programmes for HSS that applies an 'effectiveness-implementation hybrid' approach, blending components of study designs that evaluate the effectiveness of interventions on patient outcomes, with study designs that focus on evaluating the effectiveness of HSS interventions on implementation outcomes.(41) This methodology allows ASSET to understand both clinical and implementation effectiveness as well as contextual factors influencing implementation outcomes, including the potential sustainability of the different HSS interventions.

The ASSET programme includes two phases of implementation research including the pre-implementation phase, and piloting and rolling implementation phase. The *pre-implementation phase* aims to understand requirements for HSS through the evaluation of the structure and function of the health system (such as arrangements for financing, governing, and delivering care, and implementation strategies) (39) that may limit the potential to deliver evidence-informed and person-centred care. At the end of this phase, each work package use research findings to select a set of HSS interventions.

For peer review only - http://bmjopen.bmj.com/site/about/guidelines.xhtml

The *piloting and rolling implementation phase* initially pilot the interventions to identify factors that influence the implementation of the proposed interventions. After adjusting the initial programme theory and set of HSS interventions, an adaptive and iterative process is used to test the effectiveness of the set of HSS interventions on both clinical and implementation outcomes. The influence of context on the effectiveness of the HSS interventions delivering evidence-informed care is also assessed.(40) To date, ASSET has completed the pre-implementation phase of the programme and is now starting the piloting and rolling implementation phase of research. ASSET-ImplemenER, the implementation science theme within ASSET, embeds robust methodology within and across the different work packages to help ensure ASSET meet its overall aim and objectives.

Aim and objectives

The overall aim of the ASSET-ImplementER theme is to advance our understanding of how to design and evaluate HSS interventions using a systems level approach informed by implementation science, across different health systems and contexts. In doing so, we expect to achieve two equally important objectives for HSS in resource-poor contexts in LMICs: (1) advance our understanding of what HSS interventions work, for whom and how; and (2) improve implementation science methodologies to design and evaluate HSS interventions within LMIC settings.

The following specific objectives will be addressed within the ASSET-ImplementER theme:

Pre-implementation phase

1. Contrast and compare the contextual determinants identified by each work package that influence the effectiveness of evidence-informed care;
2. Contrast and compare HSS interventions selected by each work package that have the potential to improve the effectiveness of evidence-informed care;

For peer review only - http://bmjopen.bmj.com/site/about/guidelines.xhtml

3. Determine how and why the investigators from the different work packages selecte the HSS interventions and implementation outcomes and compare findings between ASSET work-packages.

Piloting and rolling implementation phase

4. Contrast and compare findings between the different work-packages on how context influences the delivery of the selected HSS interventions on standardised implementation outcomes measures for acceptability, appropriateness and feasibility;
5. Evaluate the usefulness and utility of the selected implementation science methods/frameworks in achieving a systems level approach in the evaluation of HSS interventions in resource poor contexts and provide suggestions for future applications of implementation science to HSS.

For peer review only

METHODS

Setting

ASSET is working on three care platforms: 1) integrated primary care; (2) maternal and newborn care, and; 3) surgical care, across four sub-Saharan African countries: Ethiopia, Sierra Leone, South Africa, and Zimbabwe. Within the three care platforms are eight work-packages (Table 1).

Table 1: Description of the ASSET work-packages for the different healthcare platforms; implementation research cuts across all of them (ASSET-ImplementER theme)

**Healthcare platform**	**Country**	**Specific work-package (WP)**	ASSET-ImplementER
**Primary health care for the integrated treatment of chronic conditions**	Ethiopia	WP1. Primary care for integrated person-centred continuing care of persons with chronic non-communicable diseases including diabetes and hypertension, comorbid with common mental disorders.	
	South Africa	WP4. Promoting person-centred TB care.<br>WP5. Integrated primary palliative care for persons with chronic lung disease.	
	Zimbabwe	WP8. Primary care for integrated treatment of persons with chronic non-communicable diseases including diabetes and hypertension, comorbid with common mental disorders.	

For peer review only - http://bmjopen.bmj.com/site/about/guidelines.xhtml

Healthcare platform	Country	Specific work-package (WP)	ASSET-ImplementER
**Maternal and newborn care**	Ethiopia	WP2. Integrated, person-centred and high-quality maternal and newborn care across the antenatal, intrapartum, delivery and neonatal continuum. Psychosocial care for intimate partner violence is nested within this work package.	
	South Africa	WP6. Integrated psychosocial care/support for perinatal women experiencing depression or anxiety or exposed to domestic violence.	
**Surgical care**	Ethiopia	WP3. Increasing access and quality of surgical and dental care.	
	Sierra Leone	WP7. Increasing access to quality, equitable and affordable surgical care.	

Design

ASSET-ImplementER will be embedded within the timelines for ASSET (2017 – 2022). The work-packages use mixed-methods throughout ASSET to select HSS interventions in the pre-implementation phase and evaluate the intervention through 'effectiveness-implementation hybrid' designs in the piloting and rolling implementation phase. Hybrid designs are essential with implementation research as they blend the components of study designs used to evaluate clinical effectiveness, with those of implementation study designs that focus on the evaluation of the influence of context on the effectiveness of HSS interventions.(41)

Throughout ASSET, the ASSET-ImplementER stream uses mixed methods including workshops, semi-structured interviews and documentary analyses, to standardise, record and synthesise findings from the implementation component of the different work-packages. Findings include information from the different frameworks such as context, intervention, selected HSS interventions, and implementation outcomes. Figure 1 describes the flow of methods for the ASSET-ImplementER theme.

To standardise methods and facilitate cross-site comparisons across ASSET, work-packages select contextual and behavioural determinants and implementation outcomes from a defined set implementation science frameworks. These frameworks are relevant to the programme as a whole, yet at the same time account for the specific characteristics of the different work-packages. Table 2

For peer review only

describes the frameworks that the various work-packages use and how this is relevant to the overall ASSET programme.

Figure 1: Flow of methods for the ASSET-ImplementER theme

Table 2: Selected implementation science frameworks and theories used within ASSET-ImplementER

Framework	Framework type and description	Relevance to ASSET
**Consolidated framework for Implementation Research (CFIR)**	The CFIR is a determinant framework that Includes five domains (inner setting, outer setting, intervention characteristics, characteristics of individuals involved, and the processes of implementation).(42) Within the five domains, are 37 constructs that can behave as a barrier and/or enabler to the implementation of an intervention	The appeal of the CFIR framework is the broad range of constructs that apply not only to individual characteristics, but also structural characteristics of the health care system as well as characteristics of the intervention itself. (42)
**Context and Implementation of Complex Interventions (CICI)**	The CICI is both a determinant and evaluation framework that contains three dimensions (context, implementation and setting) that interact with the intervention. The contextual dimension consists of seven contextual domains (i.e. geographical, epidemiological, socio-cultural, socio-economic, ethical, legal, political) with interact with one another at the micro, meso and macro levels.(43)	The appeal of this framework is the emphasis on the external contextual elements such as sociocultural, socioeconomic and political determinants. These determinants are particularly relevant in low-resource contexts.<br><br>This framework also explicitly addresses complexity required with the design and evaluation of HSS interventions, through the evaluation of the interaction of context (including leadership elements), implementation and setting dimensions with the intervention dimension, at the micro, meso, and macro levels.
**Theoretical Domains Framework (TDF)**	The implementation of evidence-informed interventions is dependent on changing multiple behaviours of different people.(44) where the use of theory to inform behaviour change interventions has been shown to improve the implementation effectiveness.(45) We will adopt the Theoretical Domains Framework (TDF) that represents a synthesis of 128 determinants of behavioural change.(23)	Many of the barriers to HSS interventions for ASSET are associated with determinants of current and desired behaviours e.g. in health worker or health service users. As an example, social norms that stigmatise common mental health conditions can act as a barrier to respectful, person-centred care. These determinants are particularly relevant to providing person-centred respectful care as well as high quality care.
**Behaviour Change Wheel**	The implementation of evidence-informed practice is dependent on effective behaviour change interventions. The Behavioural Change Wheel is framework that includes nine intervention functions that address determinants of problematic behaviours that require changing.	To ensure longer term sustainability, implementing evidence-informed practice for HSS will involve a degree of behavioural change. Once determinants of the problematic or desirable behaviours have been identified using the TDF, the BCW can be applied to help select appropriate interventions that have demonstrated effectiveness.

**Proctor et al Implementation outcome framework**	An evaluation framework that includes the following eight implementation outcomes: acceptability, adaptability, appropriateness, feasibility, fidelity, cost, penetration, and sustainability.(20)	All of these outcomes are relevant to ASSET at different time points in the implementation of the HSS interventions. As an example, the acceptability, appropriateness and feasibility of the HSS interventions are important to measure all phases of research as they can predict the longer-term sustainability of the intervention.(46)

The Consolidated Framework of Implementation Research (CFIR) is a determinant framework that is used as it provides an overview of a broad range of determinants that influence implementation effectiveness such as the inner setting (i.e. characteristics of the health facility), characteristics of the intervention (e.g. complexity and adaptability) and implementation processes (e.g. regular feedback about progress and quality of implementation).(42) The Context and Implementation of Complex Intervention (CICI) framework is another framework that we use as it offers a detailed approach to identifying determinants from the external context (e.g. sociocultural, socioeconomic, political, epidemiological, ethical, and legal) that are known to influence implementation effectiveness that are particularly relevant to LMICs.(43) It is expected that both of these frameworks will provide a detailed spectrum of determinants that are relevant to the ASSET programme. However, the frameworks are not exhaustive: any determinant identified that is not a part of either frameworks will be documented as such.

Further to contextual determinants, it is also important to understand in some detail characteristics of the users who deliver the healthcare. Changing behaviour ingrained in both individuals working within health systems and users of the health system, will help to ensure the adoption longer term sustainability of the HSS interventions. To address this, we will use the Theoretical Domains Framework (TDF), a determinant framework that brings together evidence based determinants of behaviour.(23) We also use the Behaviour Change Wheel (BCW), that explicitly maps behavioural change interventions onto determinants of behaviour identified with the TDF.(47) Each work-package

For peer review only

selects the contextual and behavioural determinants, implementation outcomes and HSS interventions from the different frameworks that are relevant to their aims and objectives. Table 3 describes the objectives of the different implementation science frameworks across the different phases of ASSET. However, identifying determinants that influence the effectiveness of HSS interventions in delivering evidence-informed practices is not enough to address complexity associated with HSS. We will therefore also explore how context influences the mechanisms introduced by the HSS interventions on implementation outcomes.

Table 3: Application of implementation science frameworks across ASSET health system strengthening interventions and research phases

Implementation framework	Pre-implementation phase	Piloting and rolling implementation phase
**CFIR, CICI frameworks will be used to identify determinants of the implementation process including barriers/drivers and contextual influences**	Identify barriers and/or enablers that may influence the delivery of high-quality care;<br><br>Identified barriers are used to select a set of relevant HSS interventions.	Evaluation of selected HSS interventions to understand whether they interact with contextual barrier and/or enabler as intended.<br><br>Monitor the influence of identified barriers on the effectiveness of the selected HSS interventions;<br><br>Understand/explain contextual determinants that influence implementation outcomes.
**TDF will be used to identify areas for behavioural change interventions to enhance implementation**	Identify determinants of behaviours that are known to influence healthcare professionals' ability to deliver high quality care;<br><br>Identified determinants are used to select relevant behaviour change interventions.	Evaluation of behaviour change interventions to determine whether they interact with relevant determinant of behaviour as intended.<br><br>Guide the evaluation of behaviours on implementation outcomes;<br><br>Understand/explain what behaviours influence implementation outcomes;
**Behaviour Change Wheel**	Used in combination with the TDF framework to help identify appropriate behavioural change interventions.	Used in combination with the TDF to help identify any additional behavioural change interventions.
**Proctor et al framework, framework to structure the evaluation of the implementation**	Identify implementation outcomes relevant to the aims and objectives of the work package.	Evaluate relevant implementation outcomes including acceptability, appropriateness, feasibility,<br><br>Ongoing assessment and adjustment of implementation outcomes at relevant stages of implementation

For peer review only - http://bmjopen.bmj.com/site/about/guidelines.xhtml

For peer review only

For peer review only - http://bmjopen.bmj.com/site/about/guidelines.xhtml

Measures and taxonomies

Implementation outcomes

To evaluate the effectiveness of the implementation efforts, ASSET uses a combination of implementation outcomes as defined by the framework by Proctor et al.(20) The selected implementation outcomes are aligned with the aims and objectives for the different work-packages.

Many measures used to evaluate implementation outcomes have not been validated, making it difficult to compare the effectiveness of alternative interventions both within and between studies.(46) However, three implementation outcomes measures including Acceptability of Intervention Measure (AIM), Intervention Appropriateness Measure (IAM), and Feasibility of Intervention Measure (FIM), have demonstrated promising psychometric properties in high-income settings.(46) Work-packages test the applicability of these measures in low-resourced settings by adapting them to their specific context. Each measure includes four questions measured on a Likert scale (completely disagree, disagree, neither agree nor disagree, agree, completely agree). Questions for each measure will be translated and back translated to ensure linguistic and cross-cultural equivalence. The questions will then be reviewed and cognitive testing with local participants performed prior to the tools being used.

Taxonomies

Initially we use the EPOC taxonomy for HSS interventions to label the different interventions selected by work-packages.(39) If an appropriate intervention is not included in this taxonomy, work-packages use the ERIC taxonomy for implementation strategies to label the intervention.(36) To help alleviate the ambiguity in reporting implementation strategies and HSS interventions, we also report any important differences and similarities between the two taxonomies.

For peer review only - http://bmjopen.bmj.com/site/about/guidelines.xhtml

To provide consistency in labelling of the behavioural change interventions, we use the taxonomy of behavioural change interventions that is also aligned with the TDF and the BCW.(48) However, there are important limitations to applying the TDF and BCW to help select interventions that improve the quality of people-centred care that also address issues such as illness-related stigma. As an example, these behavioural change methods were not specifically designed to address the issues surrounding implementation research in low-resource setting such as stigma, that ASSET is focusing on. As such the behavioural change interventions in this taxonomy may not be appropriate. To address this issue and similar issues with the other taxonomies, where the HSS interventions, quality improvement strategy or behavioural change intervention is not described in any taxonomy, we clearly describe the intervention and highlight the issue with the selected taxonomy not describing our intervention.

Reporting frameworks

To ensure consistent and accurate reporting of implementation studies, work-packages will apply the Standards for Reporting Implementation Studies (StaRI).(49)

Data collection procedures

Data collection in the pre-implementation phase

Workshops

At the end of the pre-implementation phase of ASSET, a two-day workshop is held involving relevant participants from all work-packages (i.e. principal investigators, co-investigators, field staff, and research assistants). The objectives of the workshop are to: (1) standardise the implementation methods being applied across the different work-packages, and; (2) collect data from each work-package on contextual and behavioural determinants associated with the delivery of high-quality evidence-informed care and the associated HSS interventions and behavioural change interventions that were selected to help deliver this care. Findings from this exercise will help to address our first

For peer review only

and second objectives of contrasting and comparing contextual and behavioural determinants between the different work-packages and the selected HSS interventions.

Using data collection tools that identify and describe determinants of the implementation process, participants within the different work-packages apply findings from the formative research to identify and record barriers and/or enablers to the delivery of high-quality care. Participants then use the selected determinants to theorise how potential HSS interventions described in the EPOC taxonomy, can assist in delivering high-quality evidence-informed care relevant to their work package. Specifically, work–packages theorise and describe the mechanisms behind how the different determinants interact to produce a consequence (i.e. poor quality of care, delayed access to care, loss to follow-up, lack of people centred care). Similar data collection methods and forms are applied to label and record determinants of behaviours that influence the delivery of evidence-informed care. The identified determinants are then be used to select and record behavioural change interventions. Templates for the workshops can be found in Appendices 1 and 2.

Interviews & focus groups

To understand our third objective of the pre-implementation phase of ASSET (i.e. how contextual determinants influence investigators in selecting HSS interventions, implementation outcomes), the workshop is followed-up with semi-structured virtual interviews involving investigators from the different work-packages. The purpose of the workshop is to help understand the 'how and why', which led them to select the HSS interventions and associated strategies to facilitate their implementation (or virtual focus groups if more feasible for country teams). The same interviews/focus groups are also used to review and finalise the findings from the workshop and to understand if there are any gaps in evidence that will require the review any additional documentation. We anticipate including a minimum of two investigators from each ASSET work-package (16 investigators minimum sample) to

For peer review only - http://bmjopen.bmj.com/site/about/guidelines.xhtml

reach saturation of the thematic areas that emerge from their responses. A guide for the interviews can be found in Appendix 3.

Data collection in the piloting and rolling implementation phase

Workshops

Throughout the piloting and rolling implementation phase, workshops are held with each work package team, involving relevant participants (principal and co-investigators, field staff, research assistants, and PhD students). The purpose of the workshops is to guide investigators on how to design the data collection tools including interview guides, using the implementation science determinant frameworks, HSS taxonomies and implementation outcomes. In doing so, we hope to ensure findings are standardised and therefore comparable across work-packages.

At the end of the piloting and rolling implementation phase, a separate workshop will be held with each of different work-package teams to feedback the following information collected using the data collection tools: (1) the effectiveness of HSS interventions on standardised implementation outcomes, (2) the influence of context on the effectiveness of HSS intervention in delivering evidence-informed care, and (3) the direct influence of context on the mechanisms introduced by the intervention to produce change. Participants are strongly encouraged to also theorise how the identified determinants interact with one another to produce mechanisms and the identified outcomes. It is through these workshops that we will be able to standardise the labelling of contextual and behavioural determinants to allow cross site comparisons on their influence of the standardised implementation outcomes (AIM, IAM, FIM) between the different work-packages. (objective 4)

At the end of the piloting and rolling implementation phase, another workshop lasting one day and involving investigators from all work-packages is used to reflect on the different implementation science methodologies and suggest improvements for further use. Specifically, we seek investigators'

For peer review only - http://bmjopen.bmj.com/site/about/guidelines.xhtml

opinions about the extent to which different frameworks capture the complexity of how the health system impacts on the overarching problem each work-package is trying to address. Again, we anticipate including a minimum of two investigators from each ASSET work-package (16 investigators minimum sample) to reach saturation of the thematic areas that emerge from their responses. All meetings will be audio-recorded with descriptive notes of discussion. Findings from these workshops are used to suggest how frameworks can be adapted to capture this complexity (objective 5).

Surveys

In work-packages that had available research capacity (WP 1, 2, 5, 6, 8), staff delivering the interventions (i.e. nurses, community health workers) are asked to complete the linguistically adapted tools for the three outcome measures, including acceptability (AIM), appropriateness (IAM) and feasibility (FIM).(46) Approximately 60 staff members from each of the participating work-packages contribute to these surveys. A subset of these participants are then interviewed to discuss the usefulness of the data collection tools. In particular, we review methods to capture the influence of context on both the effectiveness of the HSS interventions in influencing implementation outcomes as well as the influence of context on influencing the mechanisms introduced by the HSS interventions on implementation outcomes. Participants are also questioned on recommendations to improve our ability to capture the influence of context on implementation outcomes (Objective 5).

Documentary analysis

Theory-of-Change (ToC) methodology is a participatory approach involving key stakeholders that allows the articulation of the 'theory' of how a complex interventional programme will work in reality, describing the necessary interventions to bring about the change, as well as the assumptions inherent to the programme and importantly the context of implementation.(50) ASSET work-packages are developing ToCs to support their implementation and evaluation planning. ToCs are effectively programme theories, contextualised within each one of the ASSET work-packages, offering an

For peer review only - http://bmjopen.bmj.com/site/about/guidelines.xhtml

overview of how the selected HSS interventions are theorised to achieve specific implementation outcomes. ToCs also include information on key assumptions and work-package context. Each work package including all relevant stakeholders, develops and initial ToC in the pre-implementation phase. This programme theory is then adapted throughout the phases. We examine whether and how the ToCs align with findings from both the pre-implementation and piloting and rolling implementation phases of the different work-packages including determinants identified using the implementation science frameworks outlined in Table 3. We will also use ToC to better articulate how context influences mechanisms introduced by the HSS interventions described by each work package (Objective 4).

For peer review only

Data Analysis

Psychometric assessment of outcome measures AIM, IAM and FIM

The outcome measures AIM, IAM and FIM that have demonstrated promising psychometric properties in high-income countries, are tested for similar properties including substantive and discriminant content validity (the extent to which a measure is judged to be reflective of a construct of interest) and inter-item consistency (extent to which scale items are scored in a similar manner) by the different work-packages (45) to determine their relevance in low-resource settings. Measures are adapted to the local context, translated and back translated.

To test for substantive validity (extent to which a measure is reflective of the construct of interest) and discriminant content validity (extent to which a measure that are not supposed to be related are actually unrelated,) different cadres of workers who are responsible for delivering the intervention in the different work-packages assign 31 items reflecting the three constructs, to each of the three constructs and rate their confidence in the assignments in order test. The Wilcoxon one-sample signed rank test or t-test (as appropriate) is used to determine whether items measured the intended construct, or whether items measured a combination of constructs. Hochberg's correction is used to

For peer review only - http://bmjopen.bmj.com/site/about/guidelines.xhtml

correct for multiple tests.(51) Intraclass correlation coefficients (ICCs) using a two-way mixed effects model to assess the level of agreement in item assignments among all participants, and also within key stakeholder groups, across 31 items and for each construct.(52) The same data are also used to assess the factorial validity of the three measures, initially through exploratory factor analyses.(46) We assess inter-item consistency by computing Cronbach alpha for each of the four-item scales. For each measure, we also calculate means and standard deviations. Higher scores represent more favourable responses. If the measures demonstrate adequate psychometric properties, they are used to facilitate cross-site comparisons across the different work-packages.

Data collected by ASSET-ImplementER

For each work package, a thematic analysis is used to analyse the qualitative data collected in semi-structured interviews and workshops that identify key issues pertinent to using implementation science frameworks and implementation outcome measures for HSS in low-resourced contexts.

Analysis and synthesis of data collected by different work-packages

At the end of each phase of research, data is collected from each of the work-packages (i.e. contextual and behavioural determinants, mechanisms, selected HSS interventions and implementation outcomes) and entered into a template created in Excel software. As an example, each work-package records information on identified barriers/enablers, associated data source, relevant implementation science framework, EPOC HSS interventions, and implementation outcomes. Table 4 demonstrates an example of the Excel template.

Table 4: Sample template used to synthesise findings from the workshops and interviews

Determinant	Data source for determinants	Implementation science framework	EPOC health system strengthening intervention	ERIC implementation strategy	Behavioural change intervention
Illness-related stigma	Focus-group discussion with key stakeholders	CICI/TDF	Educational meetings with health care providers, educational materials distributed to patients and clinicians	Education and training	Motivate health workers

For peer review only - http://bmjopen.bmj.com/site/about/guidelines.xhtml

We adapt the matrixed multiple case study approach to analyse and synthesise the data we record in the templates.(53) This method facilitates comparisons between relevant work-packages, organising, analysing and presenting common and heterogeneous findings across implementation sites. Such an approach also aims to create generalisable knowledge regarding what and how local factors influence implementation. The matrixed multiple case study approach uses a combination of quantitative and qualitative methods that will allow us to identify associations between specific implementation processes and contextual factors on the one hand, and implementation outcomes on the other.

Initially, data are analysed separately for each work package. Quantitative data are analysed using means and proportions. Given the heterogeneous nature of the care platforms and associated work-packages, context, and selected HSS interventions, it will not be useful to compare quantitatively the influence of context on the effectiveness of HSS interventions on implementation outcomes between the work-packages. Instead, we use a qualitative approach that aims to understand why implementation outcomes were similar or different by describing the associated HSS interventions and contextual and behavioural determinants.

Results from the matrixed multiple case study approach as well as findings from the interviews and the final workshop will be triangulated to identify points of convergence and divergence between different work-packages. Using these methods, we analyse how each work-package defined and operationalised different implementation science constructs (e.g. context, intervention mechanisms, HSS interventions, behaviour change techniques), and how implementation theories and frameworks were used to support specific HSS interventions throughout implementation. The final part of our analysis integrates these elements to offer an overarching understanding of how implementation science frameworks have been operationalised across all ASSET work-packages and whether the use of these frameworks offered added utility (from a design, implementation or evaluation perspectives).

For peer review only - http://bmjopen.bmj.com/site/about/guidelines.xhtml

Patient public involvement

Patients and the public were not involved in the designing/writing protocol for this protocol. However, extensive participatory methods that involve both the patients and public will be used by the individual work-packages to design, select and evaluate the HSSIs for ASSET.

Ethics

The ASSET-ImplementER theme is considered minimal risk as we only interview researchers involved in the different work-packages. To this effect we have received approval from King's College London Ethics Committee for research that is considered minimal risk (Reference number: MRA-20/21-21772).

Discussion

To our knowledge, ASSET-ImplementER is one of the first global health implementation research programmes that attempts to standardise methodologies to design and evaluate HSS interventions across different healthcare platforms and settings. In doing so, we expect to improve our understanding of what HSS interventions work for whom, by creating a compilation of HSS interventions used by the different work-packages, the associated barriers, and the effectiveness on implementation outcomes.

ASSET-ImplementER will also attempt to develop novel insights into how we can improve implementation science methodologies for designing implementation research on HSS in LMICs. Importantly, there is a lack of standardisation in not only the terminology used to describe implementation research and HSS, but also the methods applied to this research. By labelling contextual and behavioural determinants using established frameworks and testing psychometric properties of implementation outcome measures that have demonstrated similar properties in high-

For peer review only - http://bmjopen.bmj.com/site/about/guidelines.xhtml

income countries, we hope to improve our ability to compare the effectiveness of different HSS interventions. Importantly, many researchers find the plethora of implementation science frameworks and theories overwhelming and difficult to apply in practice. Through interviews with different stakeholders, we hope to improve methods for HSS in LMICs including our ability to learn how to best apply and simplify frameworks across different health systems.

ASSET is explicitly addressing scalability through extensive participatory work, especially in the ToC workshops to develop and refine a programme theory. As an example, ToC workshops engage a wide range of stakeholders including policy makers that has a focus on ensuring scalability. Additionally, the different work packages embed the different HSS interventions within the health systems, using existing healthcare staff, facilities and policies. This approach is in agreement with recommendations for ensuring scalability.(54)

There are limitations to ASSET-ImplementER. Although our programme is theory-informed through the use of ToC workshops and implementation science determinant frameworks, we have not used implementation science theories or other middle-range theories to guide the design of ASSET as a programme. Indeed, many people criticise determinant frameworks (which we apply heavily within ASSET) as being `a' theoretical. Nevertheless, given many of the work-packages teams were unfamiliar with implementation science methods at the time of ASSET set-up, we feel this is a pragmatic approach to applying a theory-informed approach to HSS. To mitigate the effects of this static approach to our research, we work with the different work-packages throughout the different phases of research, to theorise and conceptualise how the selected determinants interact with mechanisms introduced by the selected HSS on implementation outcomes.

The coronavirus disease 2019 (COVID-19) pandemic has impacted on the pre-implementation phase of ASSET that will have consequences in what can be achieved in the longer term. In some cases,

For peer review only - http://bmjopen.bmj.com/site/about/guidelines.xhtml

work-packages experienced disruptions as key research activities such as focus group meetings, interviews with patients could not take place in person. Other issues include principal investigators for the different work packages being seconded away to COVID-19 related activities.

ASSET-ImplementER applies a system-level approach to both the design and evaluation of HSS interventions for the ASSET programme. Although there are limitations to our approach, we expect to begin advance our understanding of what HSS work for whom, and how. It is also hoped that we will start to address the issues in understanding the complexity surrounding how to effectively strengthen health systems in resource-poor contexts within LMICs.

Figure 1: Flow of methods for the ASSET-ImplementER theme

Appendix 1: Template for the workshop at the end of the pre-implementation phase of ASSET to identify determinants for the delivery of high-quality care

Appendix 2: Template for the workshop at the end of the pre-implementation phase to identify health system strengthening intervention

For peer review only

References

1. United Nations. Transforming our world: The 2030 Agenda for Sustainable Development. 2015 Accessed on: 17/October/2019. http://www.un.org/ga/search/view_doc.asp?symbol=A/RES/70/1&Lang=E. 2015.
2. Sachs J. From Millennium Development Goals to Sustainable Development Goals. The Lancet. 2012;379(9832):2206-11.
3. United Nations. Transforming our world: the 2030 Agenda for Sustainable Development http://www.un.org/ga/search/view_doc.asp?symbol=A/RES/70/1&Lang=E. 2015.
4. Bickler SN WT, Kassebaum N,. In: Debas HT, Donkor P, Gawande A, et al., editors. Essential Surgery: Disease Control Priorities, Third Edition (Volume 1). Washington (DC): The International Bank for Reconstruction and Development / The World Bank; 2015 Apr 2. Chapter 2. Available from: https://www.ncbi.nlm.nih.gov/books/NBK333518/ doi: 10.1596/978-1-4648-0346-8_ch22015.
5. Kruk M, D Gage A, Joseph N, et al. Mortality due to low-quality health systems in the universal health coverage era: a systematic analysis of amenable deaths in 137 countries. Lancet. 2018;392.
6. Ng M, Misra A, Diwan V, et al. An assessment of the impact of the JSY cash transfer program on maternal mortality reduction in Madhya Pradesh, India. Glob Health Action. 2014;7:24939.
7. Powell-Jackson T, Mazumdar S, Mills A. Financial incentives in health: New evidence from India's Janani Suraksha Yojana. J Health Econ. 2015;43:154-69.
8. World Health Organization. Everybody business : strengthening health systems to improve health outcomes : WHO’s framework for action. Geneva, Switzerland; 2007.
9. Witter S, Palmer N, Balabanova D, et al. Health system strengthening—Reflections on its meaning, assessment, and our state of knowledge. The International journal of health planning and management. 2019;34(4):e1980-e9.
10. De Savigny D, Adam T. Systems thinking for health systems strengthening. Alliance for Health Policy and Systems Research,WHO. 2009.
11. Chee G, Pielemeier N, Lion A, et al. Why differentiating between health system support and health system strengthening is needed. The International journal of health planning and management. 2013;28(1):85-94.
12. World Health Organization. Everybody business: strengthening health systems to improve health outcomes: WHO's framework for action. Geneva. WHO. 2007.
13. Borghi J, Chalabi Z. Square peg in a round hole: re-thinking our approach to evaluating health system strengthening in low-income and middle-income countries. BMJ Global Health. 2017;2(3):e000406.
14. Peters DH, Adam T, Alonge O, et al. Implementation research: what it is and how to do it. BMJ. 2013;347.
15. Peters D TN, Adam T,. Implementation research in health: a practical guide. Alliance for Health Policy and Systems Research, World Health Organization, 2013.
16. Theobald S, Brandes N, Gyapong M, et al. Implementation research: new imperatives and opportunities in global health. The Lancet. 2018;392(10160):2214-28.
17. Eccles M, Mittman B. Welcome to Implementation Science. Implementation Science. 2006;1(1):1.

For peer review only - http://bmjopen.bmj.com/site/about/guidelines.xhtml

18. Tabak R, Khoong E, Chambers D, et al. Bridging research and practice: models for dissemination and implementation research. Am J Prev Med. 2012;43(3):337-50.
19. Nilsen P. Making sense of implementation theories, models and frameworks. Implementation Science. 2015;10(1):53.
20. Proctor E, Silmere H, Raghavan R, et al. Outcomes for Implementation Research: Conceptual Distinctions, Measurement Challenges, and Research Agenda. Administration and policy in mental health. 2011;38(2):65-76.
21. Glasgow RE, McKay HG, Piette JD, et al. The RE-AIM framework for evaluating interventions: what can it tell us about approaches to chronic illness management? Patient Educ Couns. 2001;44.
22. Craig P, Di Ruggiero E, Frohlich KL. Chapter 3, Taking account of context in the population health intervention research process. Canadian Institutes of Health Research (CIHR) - National Institute for Health Research (NIHR). Southampton (UK): NIHR Journals Libary; 2018.
23. Atkins L, Francis J, Islam R, et al. A guide to using the Theoretical Domains Framework of behaviour change to investigate implementation problems. Implementation Science. 2017;12(1):77.
24. Rogers PJ. Using Programme Theory to Evaluate Complicated and Complex Aspects of Interventions. Evaluation. 2008;14(1):29-48.
25. Adam T, Hsu J, de Savigny D, et al. Evaluating health systems strengthening interventions in low-income and middle-income countries: are we asking the right questions? Health Policy Plan. 2012;27 Suppl 4:iv9-19.
26. Alonge O, Rodriguez D, Brandes N, et al. How is implementation research applied to advance health in low-income and middle-income countries? BMJ Global Health. 2019;4(2):e001257.
27. Ridde V. Need for more and better implementation science in global health. BMJ Global Health. 2016;1(2):e000115.
28. Ridde V, Pérez D, Robert E. Using implementation science theories and frameworks in global health. BMJ Global Health. 2020;5(4):e002269.
29. Pantoja T, Opiyo N, Lewin S, et al. Implementation strategies for health systems in low-income countries: an overview of systematic reviews. The Cochrane database of systematic reviews. 2017;9(9):CD011086-CD.
30. Colquhoun H, Leeman J, Michie S, et al. Towards a common terminology: a simplified framework of interventions to promote and integrate evidence into health practices, systems, and policies. Implementation Science. 2014;9(1):781.
31. McKibbon K, Lokker C, Wilczynski N, et al. A cross-sectional study of the number and frequency of terms used to refer to knowledge translation in a body of health literature in 2006: a Tower of Babel? Implementation Science. 2010;5(1):16.
32. Eccles MP, Mittman BS. Welcome to Implementation Science. Implementation Science. 2006;1(1):1.
33. Shojania K, Grimshaw J. Evidence-Based Quality Improvement: The State Of The Science. Health Affairs. 2005;24(1):138-50.
34. Cotterill S, Knowles S, Martindale AM, et al. Getting messier with TIDieR: embracing context and complexity in intervention reporting. BMC Medical Research Methodology. 2018;18(1):12.

For peer review only

35. Hoffmann T, Glasziou P, Boutron I, et al. Better reporting of interventions: template for intervention description and replication (TIDieR) checklist and guide. BMJ 2014;348:g1687.
36. Powell B, Waltz Thomas, Chinman Matthew, et al. A refined compilation of implementation strategies: results from the Expert Recommendations for Implementing Change (ERIC) project. 2015;10(1):21.
37. Booth A, Moore G, Flemming K, et al. Taking account of context in systematic reviews and guidelines considering a complexity perspective. BMJ Global Health. 2019;4(Suppl 1):e000840.
38. Shojania KG MK, Wachter RM,. Closing the Quality Gap: A Critical Analysis of Quality Improvement Strategies (Vol. 1: Series Overview and Methodology). Rockville (MD): Agency for Healthcare Research and Quality (US); 2004 Aug. (Technical Reviews, No. 9.1.) Available from: https://www.ncbi.nlm.nih.gov/books/NBK43908/. 2004.
39. Effective Practice and Organisation of Care (EPOC). The EPOC taxonomy of health systems interventions. EPOC Resources for review authors. . Oslo: Norwegian Knowledge Centre for the Health Services; 2016.
40. ASSET Health System StrEngThening in Sub Saharan Africa 2020 [The web page for the NIHR funded ASSET programme].
41. Curran G, Bauer M, Mittman B, et al. Effectiveness-implementation hybrid designs: combining elements of clinical effectiveness and implementation research to enhance public health impact. Medical care. 2012;50(3):217-26.
42. Damschroder L, Aron D, Keith R, et al. Fostering implementation of health services research findings into practice: a consolidated framework for advancing implementation science. Implementation Science. 2009;4(1):50.
43. Pfadenhauer L, Gerhardus A, Mozygemba K, et al. Making sense of complexity in context and implementation: the Context and Implementation of Complex Interventions (CICI) framework. Implementation Science. 2017;12(1):21.
44. Grol R, Grimshaw J. From best evidence to best practice: effective implementation of change in patients' care. Lancet. 2003;362(9391):1225-30.
45. Noar S, Zimmerman R. Health Behavior Theory and cumulative knowledge regarding health behaviors: are we moving in the right direction? Health Education Research. 2005;20(3):275-90.
46. Weiner B, Lewis C, Stanick C, et al. Psychometric assessment of three newly developed implementation outcome measures. Implementation science : IS. 2017;12(1):108-.
47. Michie S, van Stralen M, West R. The behaviour change wheel: A new method for characterising and designing behaviour change interventions. Implementation Science. 2011;6(1):42.
48. Michie S, Richardson M, Johnston M, et al. The behavior change technique taxonomy (v1) of 93 hierarchically clustered techniques: building an international consensus for the reporting of behavior change interventions. Annals of behavioral medicine : a publication of the Society of Behavioral Medicine. 2013;46(1):81-95.
49. Pinnock H, Epiphaniou E, Sheikh A, et al. Developing standards for reporting implementation studies of complex interventions (StaRI): a systematic review and e-Delphi. Implementation Science. 2015;10(1):42.
50. Vogel I. Review of the use of theory of change in international development. UK: Department for International Development, 2012.

For peer review only - http://bmjopen.bmj.com/site/about/guidelines.xhtml

51. Hochberg Yosef. A sharper Bonferroni procedure for multiple tests of significance. Biometrika. 1988;75(4):800-2.
52. Dixon D, Pollard B, Johnston M. What does the chronic pain grade questionnaire measure? Pain. 2007;130(3):249-53.
53. Kim B, Sullivan J, Ritchie M, et al. Comparing variations in implementation processes and influences across multiple sites: What works, for whom, and how? Psychiatry Research. 2019:112520.
54. Barker P, Reid A, Schall M. A framework for scaling up health interventions: lessons from large-scale improvement initiatives in Africa. Implementation Science. 2016;11(1):12.

Declarations

Competing interests: None declared

Patient consent: Protocol paper so consent not applicable

Data sharing statement: No additional data available as a protocol paper

Ethics: Approval from King's College London Ethics Committee for research that is considered minimal risk (Reference number: MRA-20/21-21772).

Funding: The study is funded by the National Institute of Health Research (NIHR) Global Health Research Unit on Health System Strengthening in Sub-Saharan Africa, King's College London (GHRU 16/136/54) using UK aid from the UK Government to support global health research. GT, WG and NS' research is also supported by the National Institute for Health Research (NIHR) Applied Research Collaboration (ARC) South London at King's College Hospital NHS Foundation Trust. GT and NS are members of King's Improvement Science, which offers co-funding to the NIHR ARC South London and is funded by King's Health Partners (Guy's and St Thomas' NHS Foundation Trust, King's College Hospital NHS Foundation Trust, King's College London and South London and Maudsley NHS Foundation Trust), Guy's and St Thomas' Charity and the Maudsley Charity. NS' research is further supported by the ASPIRES research programme (Antibiotic use across Surgical Pathways - Investigating, Redesigning and Evaluating Systems), funded by the Economic and Social Research Council. GT also receives support from the National Institute of Mental Health of the National Institutes of Health under award number R01MH100470 (Cobalt study) and by the UK Medical Research Council in relation the Emilia (MR/S001255/1) and Indigo Partnership (MR/R023697/1)

For peer review only - http://bmjopen.bmj.com/site/about/guidelines.xhtml

awards. The views expressed are those of the authors and not necessarily those of the NHS, the NIHR, the ESRC, the MRC, the charities or the Department of Health and Social Care. CH receives support from AMARI as part of the DELTAS Africa Initiative (DEL-15-01).

Authors contributions:

NSeward wrote the first and subsequent drafts of the paper

NSeward and NSevdalis conceptualised the idea for the paper

NSeward, NSevdalis, Jamie Murdoch and Charlotte Hanlon offered insight and edited all drafts of the paper

All other authors (RA, WG, RH, CL, SHK, RM, KM, MP, JS, GT, RV) edited and offered input to various drafts of the paper

For peer review only

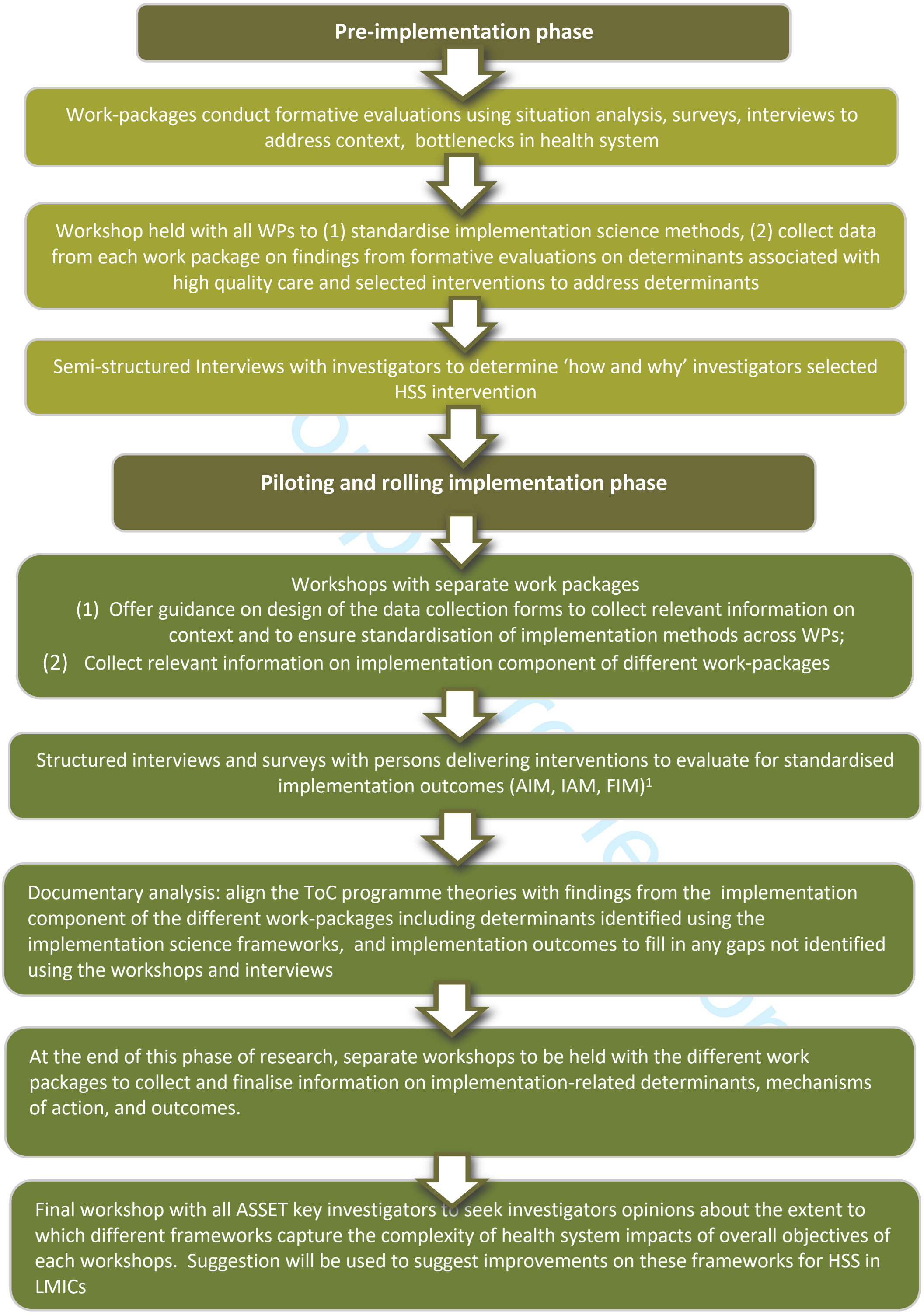

1. Acceptability of Intervention Measure (AIM), Intervention Appropriateness Measure (IAM), and Feasibility of Intervention Measure (FIM), have demonstrated promising psychometric properties in high-income settings.(38)

Figure 1: Flow of methods for the ASSET-ImplementER theme

Workshop for ASSET meeting in Addis Ababa Ethiopia, February 2020

Template for identification of contextual and behavioural determinants that can potentially influence the effectiveness of your intervention

A. Context determinant that influence the delivery of care relevant to your work package

Context refers to anything in the environment, that influences the delivery of high-quality, evidence-based care that is person-centred and respectful.

1. List all the contextual barriers/enablers identified in the diagnostic phase of your research that influenced the delivery of high-quality care.
2. For each contextual determinant listed below, theorise the proposed mechanism you think this will influence the implementation of your intervention.
3. We will be visiting each work-package to assist you in any way we can.

Table 1: Contextual determinants that can potentially influence the effectiveness of implementation efforts

Domain	Determinants	Describe how relevant determinant influences the implementation of your intervention
The geographical domain refers to the broader physical environment, landscapes and resources, both natural and transformed by humans, available at a given location. As such, it also comprises the infrastructure at a given location, which	Geography (e.g. altitude, desert, forest, water)<br><br>Climate (e.g. temperature, rainfall)<br><br>Infrastructure (e.g. water and sanitation, energy, transport)<br><br>Access to health care system	i.e. distance to health centre, rainfall, etc

For peer review only - http://bmjopen.bmj.com/site/about/guidelines.xhtml

could result in geographical isolation.		
Epidemiological: distribution of disease/conditions, the attributable burden of disease as well as determinants of needs in human populations	Demographics (life expectancy, gender, age, ethnicity, genetic factors)<br><br>Population density, fertility patterns, family size<br><br>Incidence/prevalence and severity of disease, morbidity and mortality	i.e. infectious nature of tuberculosis
Socioeconomic: This domain comprises the economic resources of a community and the access of a population to these resources. It also shows the relationship between an economy and its society.	Social or socio-economic status attributed to education, income, occupation marriage, or gender<br><br>Financial aspects (income, wealth)<br><br>Occupational aspects (employment status, working conditions)<br><br>Living conditions (housing, neighbourhood characteristics)<br><br>Determinants of needs of people directly affected by disease/condition	Example – costs of seeking care and associated childcare costs; lack of education and associated literacy influencing patients ability to seek appropriate care

For peer review only - http://bmjopen.bmj.com/site/about/guidelines.xhtml

	Access to health care system	
Sociocultural:<br><br>This domain comprises explicit and implicit behaviour patterns, including their embodiment in symbols and artefacts; the essential core of culture consists of historically derived and selected ideas and values that are shared among members of a group<br><br>This domain not only refers to the conditions in which people are born, grow, live, work and age but also embraces the social roles a human being takes in as a family member, community member or citizen and the relationships inherent to these roles.<br><br>Constructs such as knowledge, beliefs, conceptions, customs, institutions and any	Language and means of communication<br><br><br><br>Symbols, heroes, rituals<br><br>Values (e.g. evil vs. good, dirty vs. clean, dangerous vs. safe, abnormal vs. normal)<br><br>Beliefs (e.g. superstition, fate or destiny)<br><br>Religiosity and spirituality<br><br>Knowledge and perceptions (e.g. with respect to significance of health issue, options for resolving health issue)<br><br>Lifestyle (population's patterns in nutrition, smoking, substance abuse)<br><br>Discrimination<br><br>Social capital and resources available through social relationships, specifically	Example – stigma associated with TB, HIV or mental health conditions; acceptance of violence as normal; normalisation of disrespectful care

For peer review only

other capabilities and habits acquired by a group are comprised by this domain	social networks, norms of reciprocity, and trust<br><br>Social cohesion, including relational, material, and political dimensions, information exchange, networks of support, and informal social control<br><br>Historical and contemporary social power relations<br>Sociodemographic profiles<br><br>Psychosocial factors<br><br>Social and societal context<br><br>Structural social inequalities (e.g. Gender inequalities, caste system)<br><br>Community characteristics and level of coordination/involvement with community<br><br>Relevant changes over time (e.g. social changes or social movements, violence)	
Political	Political situation including political stability and absence of violence,	Political uprisings that may results in conflict, that influences the delivery of high-quality care<br>Implementation and support of appropriate guideline-based care

For peer review only - http://bmjopen.bmj.com/site/about/guidelines.xhtml

	government effectiveness, voice and accountability, control of corruption, rule of law, regulatory quality, participation, accountability, transparency, efficiency, decency, and fairness<br><br>Health Care System (e.g. governance and leadership, resources, service delivery, integration of patient's needs and perspective)<br><br>Access to health care system<br><br>Relevant changes over time (e.g. political reform*, change of government)	
Legal	Norms, values and beliefs underlying legislation<br><br>Specific legislation (e.g. patient rights, data protection)<br><br>Regulatory provisions concerning healthcare personnel and their rights and duties<br><br>Guidelines	Legislation to protect women from violence; legislation act for mental health

For peer review only

	Decision-making in care delivery<br><br>Sharing of information with indirectly affected stakeholders<br><br>Legislation<br><br>Relevant changes over time (e.g. introduction of new regulation or legislation)	
Ethical	Autonomy<br><br>Moral distress<br><br>Privacy<br><br>Conflicting interests<br><br>Morality and beliefs<br><br>Ethical principles and code of conduct	

For peer review only

B. Characteristics of the organisation implementing the intervention (i.e. primary health centre, hospital, community setting) that influence the delivery of high-quality care

The characteristics of the site where the intervention is going to be implemented is known to influence the effectiveness of implementation efforts.

Below are a list of possible characteristics of the health facility or organisation that can influence the delivery of high-quality care.

In your different work packages, please read the different questions for each of the listed determinants. If you feel the determinants influence the delivery of high-quality care, please theorise and describe the mechanisms by which you feel this occurs.

Determinant	Relevant questions	How does this influence delivery of high-quality care
Structural characteristics: These are objective organisational characteristics – e.g., the size, location, financial turnover, patient volumes, number of services and similar. These characteristics of your hospital may impact positively or negatively on the planned implementation.	How does the infrastructure of the organisation, influence the delivery of high-quality care?<br><br>How will the infrastructure influence the implementation of the intervention?<br><br>Are there any changes in infrastructure that will be required to implement the intervention?	
Networks and communications: Staff and other peer-to-peer networks and communications systems and policies within your organisation. Includes written, oral and other methods of communication.	How available is information to deliver required guideline-based care?<br>How available are people when you need help in solving a problem?<br>How are the working relationships with leaders?<br>Working relationships with influential stakeholders?<br>Working relationships with colleagues?	

For peer review only

Determinant	Relevant questions	How does this influence delivery of high-quality care
Culture: Norms, values, and basic assumptions of a given organization.	How will/does the organisation culture, influence the delivery of high-quality care or the implementation of the intervention?<br>To what extent does the organisation embrace new ideas to improve the organisation?<br>What is the culture like within the health facilities?<br>Are the health facilities innovative?<br>Are the health facilities safety and quality conscious? Or slow to respond?<br>Other cultural elements?	
Implementation climate<br>This is a specific element of organisational culture around how innovations and evidenced interventions tend to get implemented. Check the 6 elements on the left – how do they apply to your organisation?	1. Tension for change<br>2. Compatibility<br>3. Relative priority<br>4. Organisational incentives and rewards<br>5. Goals and feedback<br>6. Learning climate	
Readiness for implementation: As above, this is a cultural element specifically applied to implementing innovations. Check the 3 elements on the right – how do they apply to your organisation?	1. Leadership engagement<br>2. Available resources<br>3. Access to information and technology	
Patients' needs and resources	Is the facility aware of the needs of the patients accessing the health facility?<br><br>Does the facility have the resources to adequately address the needs of the patients?	

For peer review only - http://bmjopen.bmj.com/site/about/guidelines.xhtml

Determinant	Relevant questions	How does this influence delivery of high-quality care

C. Determinants of behaviours that influence the delivery of high-quality care

Recognising and targeting determinants of behaviours that influence the delivery of high-quality, person centred care will be an important component of the interventions for the different work packages within ASSET.

The Theoretical Domains Framework (TDF) was developed to identify determinants of specific behaviours. For this exercise we have selected behaviours associate with person-centred care or quality improvement as these are common themes across all work packages.

Once the key determinants for specific behaviours associated with person centred care or quality improvement have been identified using the TDF, there is potential to design interventions that specifically target the identified determinants.

Behaviour change techniques have also been mapped onto the TDF that allows us to identify key determinants for a particular behaviour associated the person-centred care/quality improvement and propose a set of behaviour change techniques.

We ask that each work package splits into two groups – one for person centred care and one for quality improvement. Initially we that you list below the problematic behaviours associated with either person centred care or quality improvement. Once this is complete, we ask that you identify determinants of these behaviours using the template provided in Table 5. Finally, within each work packages, identified problematic behaviours and associated determinants will be cross-checked by individuals on working on the different set of behaviours (i.e. people working on person centred care will cross check quality improvement, and vies versa)

For peer review only

Behaviours associated with person centred care and quality improvement

We have started to list behaviours associated with person-centred care in Table 3 below. Please list any additional behaviours relevant to person centred care that you feel are relevant.

Table 3: Behaviours associated with person-centred care

**Behaviours associated with person centred care**	Indicator details
Person centred communication	Patients are actively involved in discussions and decisions about their care; whether professionals encourage patients and families to express their needs, preferences and concerns; whether professionals monopolise the conversation and the extent to which patients feel engaged and valued.
Supporting self-management	This is the encouragement that professionals provide to help patients understand their central role in managing their condition and making decisions.
Supporting shared decision making	Shared decision-making involves patients and professionals communicating about potential care options, and professionals supporting patients to consider the possible consequences of options and the evidence available before arriving at informed preferences
Respectful, compassionate care	Doctors and nurses show interest in patient as a person and is asked about mental state and/or fear
Empathy, compassion, dignity	Dignity involves ensuring people know they are worthy of respect.<br>Doctors and nurses have empathy with patients' emotions and actual situation

Mapping determinants of behaviours associated with person-centred care or quality improvement

Table five lists potential determinants of problematic behaviours associated with person centred care and the quality improvement. Please work within your groups, to identify determinants of person-centred care or quality improvement for each of the behaviours identified in Table 3 or Table 4.

For peer review only - http://bmjopen.bmj.com/site/about/guidelines.xhtml

Table 5: Determinants of behaviours associated with person-centred care

Determinants of problematic behaviours	Examples	Is this determinant applicable to the relevant problematic behaviour in your work package
1.Lack of knowledge<br>(An awareness of the existence of something)	Knowledge (including knowledge of condition/scientific rationale)<br>Procedural knowledge<br>Knowledge of task environment	
2.Lack of skills<br>(An ability or proficiency acquired through practice)	Skills<br>Skills development<br>Competence<br>Ability<br>Interpersonal skills<br>Practice<br>Skill assessment	
3. Social/professional role and identity<br>(A coherent set of behaviours and displayed personal qualities of an individual in a social or work setting)	Professional identity<br>Professional role<br>Social identity<br>Identity<br>Professional boundaries<br>Professional confidence<br>Group identity<br>Leadership<br>Organisational commitment	
4. Beliefs about capabilities<br>(Acceptance of the truth, reality or validity about an ability, talent or facility that a person can put to constructive use)	Self-confidence<br>Perceived competence<br>Self-efficacy<br>Perceived behavioural control<br>Beliefs<br>Self-esteem<br>Empowerment<br>Professional confidence	

**Determinants of problematic behaviours**	**Examples**	**Is this determinant applicable to the relevant problematic behaviour in your work package**
5. Optimism<br>(The confidence that things will happen for the best or that desired goals will be attained)	Optimism<br>Pessimism<br>Unrealistic optimism<br>Identity	
6. Beliefs about Consequences<br>(Acceptance of the truth, reality, or validity about outcomes of a behaviour in a given situation)	Beliefs<br>Outcome expectancies<br>Characteristics of outcome expectancies<br>Anticipated regret<br>Consequents	
7. Reinforcement<br>(Increasing the probability of a response by arranging a dependent relationship, or contingency, between the response and a given stimulus)	Rewards (proximal/distal, valued/not valued, probable/improbable)<br>Incentives<br>Punishment<br>Consequents<br>Reinforcement<br>Contingencies<br>Sanctions	
8. Intentions<br>(A conscious decision to perform a behaviour or a resolve to act in a certain way)	Stability of intentions<br>Stages of change model<br>Transtheoretical model and stages of change	
9. Goals<br>(Mental representations of outcomes or end states that an individual wants to achieve)	Goals (distal/proximal)<br>Goal priority<br>Goal/target setting<br>Goals (autonomous/controlled)<br>Action planning<br>Implementation intention	

Determinants of problematic behaviours	Examples	Is this determinant applicable to the relevant problematic behaviour in your work package
10. Memory, attention and decision processes<br>(The ability to retain information, focus selectively on aspects of the environment and choose between two or more alternatives)	Memory<br>Attention<br>Attention control<br>Decision making<br>Cognitive overload/tiredness	
11. Environmental context and resources<br>(Any circumstance of a person's situation or environment that discourages or encourages the development of skills and abilities, independence, social competence and adaptive behaviour)	Environmental stressors<br>Resources/material resources<br>Organisational culture/climate<br>Salient events/critical incidents<br>Person × environment interaction<br>Barriers and facilitators	
12. Social influences<br>(Those interpersonal processes that can cause individuals to change their thoughts, feelings, or behaviours)	Social pressure<br>Social norms<br>Group conformity<br>Social comparisons<br>Group norms<br>Social support<br>Power<br>Intergroup conflict<br>Alienation<br>Group identity<br>Modelling	
13. Emotion<br>(A complex reaction pattern,	Fear<br>Anxiety	

For peer review only - http://bmjopen.bmj.com/site/about/guidelines.xhtml

Determinants of problematic behaviours	Examples	Is this determinant applicable to the relevant problematic behaviour in your work package
involving experiential, behavioural, and physiological elements, by which the individual attempts to deal with a personally significant matter or event)	Affect<br>Stress<br>Depression<br>Positive/negative affect<br>Burn-out	
14. Behavioural regulation (Anything aimed at managing or changing objectively observed or measured actions)	Self-monitoring<br>Breaking habit<br>Action planning	

For peer review only

Documentation of selected HSSIs and associated contextual and intervention determinant

Task: Review the different determinants selected during Thursday's workshop and match to relevant EPOC health system strengthening interventions (attached in email)

Table 1: Identified determinant and associated HSSI/Implementation strategies

Determinant	Health system strengthening intervention/ERIC implementation strategies

Documentation of selected intervention functions and associated determinants of behaviour

Please complete the questions below, each of the identified problematic behaviours identified and the associated TDF determinants.

Table 2:

Question	Description
Name of intervention	
1. What is the behaviour that you want to change?	
2. Why do you want to change it? Why is this a priority?	
3. Who is performing the behaviour?	
4. What do they need to do differently? (In what direction do you want the behaviour to change?)	
5. When do they need to do it?	
6. Where do they need to do it?	
7. How often do they need to do it?	
8. With whom do they need to do it?	
9. Which TDF domains and components are most relevant (give codes)	

For peer review only

Map the determinants of behaviour identified with the TDF, to appropriate intervention functions using the documentation provided

Please see the attached documents to facilitate this process (behaviour change wheel, TDF mapped to BCW)

Table 3: Summary of determinants of behaviours identified with the TDF, associated intervention functions and sources of behaviour

Determinants of behaviour (TDF)	Source of behaviour (COM-B)	Intervention function (BCW)
Knowledge, cognition, memory, behaviour regulation	Capability - psychological	Education, training, enablement

For peer review only

Appendix 3: Topic guide for interviews and focus group discussions with investigators from the work packages

Work package:

Participant name:

Date:

Location

A. Introduction

Interviewer to say his/her name and where they are from.

We are here to get feedback from you on how and why you selected health system strengthening interventions. The information you give us will help us to further understand how HSS interventions are selected.

While we are talking, we are recording what is said with this tape recorder so that we can remember all of the information. There are no right or wrong answers.

B. Interview guides

Interview guide to understand how and why the investigators from the different work packages selected the HSS interventions and implementation outcomes

Objective: to get feedback from the investigators from the different work packages to help understand how and why they selected the health system strengthening interventions and associated implementation outcomes.

[Start tape recording from here]

- **To assess how the HSS interventions were selected**
  - Before you began the pre-implementation phase of your research, did you have in mind what implementation strategies you would use?
  - If this was the case, did these change during the pre-implementation phase? Tell me about the process that caused this change.
  - How (what method was used) to select the HSS interventions? Tell me about the process you went through that enabled you to make the selection.
  - Were stakeholders consulted about potential health system strengthening interventions? If so, what can you describe which stakeholders were involved and how they were involved?
  - Was the Theory of Change workshop used to facilitate the selection of interventions and implementation outcomes? If so, how did you conduct the workshops to do this? If not, why did you think they were not suitable? What did you do instead?
  - Were focus group discussions used to help select the HSS interventions and implementation outcomes? What approach did you take and how did they inform the selection of HSS interventions?

For peer review only

- Were implementation science determinant frameworks used to help select the HSS intervention? Tell me about which ones you used and how you used them to inform the selection. *(Probe for how contextual determinants were identified, role of theory and how used to guide other methods such as ToC or focus groups)*
- Were implementation evaluation frameworks used to help select the HSS interventions? Tell me about which ones you used and how you used them to inform the selection. *(Probe for why and how structure of framework was suitable and how used alongside other methods)*
- Were the implementation outcomes matched with specific HSS interventions? If so, tell me about which outcomes you selected and how the HSS interventions were theorised to impact on those outcomes.
- Was the ERIC tool used to help select the HSS interventions? How was this undertaken?
- Was a literature review done to help inform the selection of HSS interventions and outcomes? In what way did previous evidence inform the selection? What issues did you face in translating previous research to the choice of intervention in your study?
- Did you use any other methods to inform your choice of intervention that we haven't discussed?

Interview guide to finalise the findings from the workshop and to understand if there are any gaps in evidence that will require the review any additional documentation.

Objective: After reviewing the findings from the different work packages on the identified determinants and health system strengthening interventions, undoubtedly there will be a few remaining questions. The purpose of this part of the interview is to raise any remaining questions and answer any questions/concerns the work packages may have.

- **We have the following questions about the determinants and HSS interventions that you have selected:**

- **Do you have any remaining questions/concerns about your selection of HSS interventions and implementation outcomes?**

[Stop tape recording when completed]

For peer review only
